# Genome-Wide Identification, Evolution, and Transcriptional Profiling of* PP2C* Gene Family in* Brassica rapa*

**DOI:** 10.1155/2019/2965035

**Published:** 2019-04-03

**Authors:** Nadeem Khan, Han Ke, Chun-mei Hu, Emal Naseri, Muhammad Salman Haider, Aliya Ayaz, Waleed Amjad Khan, Jianjun Wang, Xilin Hou

**Affiliations:** ^1^State Key Laboratory of Crop Genetics and Germplasm Enhancement, Ministry of Science and Technology/College of Horticulture, Nanjing Agricultural University, Nanjing 210095, China; ^2^New Rural Research Institute in Lianyungang, Nanjing Agricultural University, Nanjing 210095, China

## Abstract

The type 2C protein which belongs to the major group of protein phosphatases (PP2C) plays a vital role in abscisic acid (ABA) signaling and signal transductions processes. In the present study, 131* PP2C* genes were identified in total in* Brassica rapa* and categorized into thirteen subgroups based on their phylogenetic relationships. These* B. rapa* PP2C are structurally conserved based on amino acid sequence alignment, phylogenetic analysis, and conserved domains. Moreover, we utilized previously reported RNA-sequence data on various tissues (root, stem, leaf, flower, and silique), which suggests overlapping expression pattern in 29 paralogous gene pairs. The qRT-PCR validation of 15 paralogous gene pairs depicts distinct expression patterns in response to various abiotic stresses, such as heat, cold, ABA, and drought. Interestingly, stress-responsive* BraPP2C* candidate genes were also identified, suggesting their significance in stress-tolerance mechanism in* B. rapa*. The evolutionary analysis for 15 paralogous gene pairs suggested that only three pairs have the positive selection and remaining were purifying in nature. The presented results of this study hasten our understanding of the molecular evolution of the* PP2C* gene family in* B. rapa*. Thus, it will be ultimately helping in future research for facilitating the functional characterization of* BraPP2C* genes in developing the abiotic stress tolerant plants.

## 1. Introduction

In cellular signaling, the reversible protein phosphorylation catalyzed by protein kinases (PKs) and phosphatases (PPs) is known to participate in critical processes [1]. In plants, the reversible protein phosphorylation is one of the critical modification processes, which helps in the regulation of some important physiological and biochemical reactions. The PPs provide modulations of protein phosphoregulation by reversing the action of PKs. The PPs can be categorized into two major classes, such as protein tyrosine phosphatase (PTPs) and protein serine/threonine phosphatases (PSPs) based on the substrate specificity [2]. Moreover, most of the PSPs are further subcategorized into two groups; group one includes protein phosphatase 1 (PP1), PP2A, PP2B, PP4, PP5, and PP6, while group two contains protein phosphatase M (PPM), including the PP2C and also pyruvate dehydrogenase phosphatases [2].

From prokaryotes to higher eukaryotes, the PP2C are evolutionarily conserved and mostly found in archaea, bacteria, fungi, plants, and animals [3, 4]. Furthermore, it acts as a negative modulator of PKs cascades activated and then implicated in regulating stress-signaling pathways. Like PKs, PPs also share a crucial role under various stress conditions and might be pivotal at different developmental stages of plants and signal transduction processes. Particularly in* Arabidopsis *and* Brassica rapa *recently, several groups of the researcher are engaged by deciphering the involvement of different kinases against biotic and abiotic stress networks such as MAPKs [5, 6], CDPKS [7, 8], and CIPKs [9, 10]. For instance, temperature (high and low) stresses are the major environmental factor that limits crop productivity in vegetable production [11, 12]. Generally, during cold treatment in which plants adjust their metabolism are considered as cold acclimation. To date, several studies highlighted the importance of PP2C that may play pivotal roles in various processes, including both biotic-abiotic stress factors and plant development [13, 14]. In plants, PP2C members have been described as a regulator of desiccation tolerance and act as a negative regulator in the ABA-signaling pathway [15–17]. For example, in moss, the only two* PpABI1A* and* PpABI1B *belonging to subfamily A of PP2C are known to induce desiccation tolerance in vegetable and are directly involved in ABA signaling [18]. In addition, drought stress considerably affects the crop productivity with an ultimate decline of photosynthetic assimilates, due to the osmotic stress-imposed constraints. Plants can utilize adaptive strategies against drought stress, such as escape, avoidance, and tolerance [19]. The validation of maize* ZmPP2C*-*A10* by transgenic studies has confirmed its negative regulation in drought stress tolerance [20]. In particular, the two* PP2C* genes encoding ABA-insensitive mutants (abi1-1 and abi2-1) are known to partake in the various physiological processes after exposure to abiotic stimuli, including salt, drought, and freezing [21–24]. Plant development and freezing tolerance were accelerated by downregulation of* PP2CA* genes and the antisense mediation [25]. Hence, for a better understanding of protein phosphatases and their functional significance, the identification of PP2C provides a stepping stone in stress-signaling pathways.

*Brassica rapa*, a diploid species, share a complex history with model plant* Arabidopsis* and has experienced two main duplication events such as (WGD *α* and *β*) and additional one whole-genome triplication event (WGT *γ*) 13–17 million years ago (MYA) [26, 27]. Thus, the* B. rapa *due to WGT event has experienced considerable fractionation in the genome (i.e., duplicate gene loss); as a result, it presents to us an opportunity for* PP2C* genes to study its evolutionary implications. In this study, all the* PP2C* genes through various approaches were isolated from* Arabidopsis* and* B. rapa *and their phylogenetic relationships were conducted to categorize them into multiple subgroups. In addition, we comparatively analyzed* PP2C* genes from ten representative plant species, including one of the earliest angiosperm plant* Amborella trichopoda, Populus trichocarpa, Vitis vinifera, Capsella rubella, Citrus sinensis, Carica papaya, Solanum lycopersicum, Fragaria vesca, Arabidopsis lyrata, *and a bryophyte (*Physcomitrella patens*), the earliest sequenced land plant [28].

We further compared the* PP2C* genes between* B. rapa *and* Arabidopsis, *to explore and identify both shared and specific subgroups. Following the gene structure organization analysis, conserved protein motifs, cis-elements, and interaction network, we traced paralogous gene pairs and their evolutionary divergence that likely resulted in the expansion of the* PP2C* gene family. To shed a light of some critical* BraPP2C* genes on their associated indigenous functional role were further exposed to heat, cold, ABA-signaling, and drought stress conditions. Additionally, the transcriptional profiling of the* BraPP2C *genes for various tissues and qRT-PCR analysis of 15 paralogous gene pairs were analyzed and compared. In both eukaryotes and prokaryotes, the PPs are vital in both diverse signaling pathways and kinase-counteracting components. Indeed, for phosphatases, most of the functionally characterized literatures have been studied in Arabidopsis, and minimal studies have been done in crop plants. Therefore, to our knowledge, this is the first systemic reported genome-wide and transcriptional profiling of the* PP2C* genes in* B. rapa,* and it is affirmative to carry out a comprehensive study to understand the regulation of phosphatases in* B. rapa *during stress and development.

## 2. Materials and Methods

### 2.1. Data Resources for Sequence Retrieval

For identification of* PP2C* genes in* Brassica rapa*, we utilized the Plaza 4.0 database (https://bioinformatics.psb.ugent.be/plaza/) with the help of InterPro PP2C domain “IPR001932”. The* B. rapa* genome sequences were downloaded from BRAD (http://brassicadb.org/brad/) [29], and* A. thaliana* sequences were retrieved from TAIR (http://www.arabidopsis.org/). The sequences of others species were downloaded from Phytozome v12.1.6 (https://phytozome.jgi.doe.gov/pz/portal.html) [30]. The domains of obtained BraPP2C proteins were also further verified using NCBI-Conserved Domain database (https://www.ncbi.nlm.nih.gov/Structure/cdd/wrpsb.cgi) search program and SMART databases (http://smart.embl-heidelberg.de/) [31]. Those proteins which lack PP2C domain were removed from further analysis. In addition, protein sequences that were found with obvious errors in their gene length or having less than 100 lengths were eliminated.

### 2.2. Multiple Sequence Alignment and Phylogenetic Analysis

The amino acid sequences of the BraPP2C proteins were used for further investigation, and multiple sequence alignment was performed by MUSCLE [32] using MEGA 7 software with the default options [33]. The phylogenetic trees were constructed using the maximum likelihood (ML) method. To determine the reliability of resulting tree, bootstrap values of 1000 replications were performed with the Jones, Taylor, and Thornton amino acid substitution model (JTT model), while keeping the other parameters as a default.

### 2.3. Calculation of the* Ka/Ks* for BraPP2C Paralogous Gene Pairs

The* Ka/Ks* ratios were calculated for BraPP2C paralogous gene pairs using MEGA 7.0 [33]. The divergence time among the paralog pairs was calculated with the following formula: T =* Ks*/2r, where* Ks* represents the synonymous substitutions per site and r is the rate of divergence. For dicotyledonous plants, specifically* B*.* rapa*, the assumption is 1.5 synonymous substitutions per site of 10^8^ years [34].

### 2.4. Conserved Motifs, Exon-Intron Structure Analysis, and Physicochemical Parameters of BraPP2C Proteins

Conserved motif scanning of BraPP2C proteins was carried out through local MEME Suite Version 5.0.2. For this purpose, parameter settings were calibrated as follows: maximum number of motifs 10, with a minimum width of 100 and a maximum of 120. The other parameters were set as default [35]. For the exon-intron structure, we used the Gene Structure Display Server (GSDS 2.0) (http://gsds.cbi.pku.edu.cn) [36]. The physicochemical properties of the proteins, including molecular weight (MW), isoelectronic points (pI), and GRAVY values for each gene, were calculated using the ExPASY PROTPARAM tools (http://web.expasy.org/protparam/). The subcellular localization was predicted using the WOLF PSORT (https://wolfpsort.hgc.jp/) website.

### 2.5. Cis-Elements Predictions and Interaction Network of BraPP2C

Every BraPP2C promoter sequence (selected as 2000 upstream bp) was imported in Generic File Format (GFF) file from the* B*.* rapa *genome. Then, PlantCARE database (http://bioinformatics.psb.ugent.be/webtools/plantcare/html/) [37] was utilized to identify the cis-regulatory elements for promoters of each gene. The interaction network of BraPP2C proteins was constructed with the help of STRING software (https://string-db.org/).

### 2.6. Chromosomal Location and Paralogous Gene Pairs Identification of BraPP2C

The chromosomal location* BraPP2C* genes were illustrated from top to bottom concerning their position in the genome annotation using Mapchart [38]. For synteny gene analysis, the relationships were verified between the homologs of* A. thaliana* and subgenomes of* B. rapa* (LF, MF1, and MF2) obtained from BRAD (http://brassicadb.org/brad/searchSynteny.php). Furthermore, the paralogous genes were identified either selecting genes pair between LF1 and MF1 or MF1 and MF2 subgenomes of* Brassica rapa*, respectively. Circos program was applied to demonstrate the syntenic relationships among the chromosomes of* B. rapa* and* A. thaliana* [39].

### 2.7. Pearson Correlation Analyses

Pearson correlation (PCC) analysis was performed with the help of Excel 2013 to evaluate the PCC values of the RNA-seq and the paralogous genes that were used for qRT-PCR [40].

### 2.8. Plant Material and Treatments

In the present study, the germinated seeds of Chinese cabbage (Chiifu-401-42) were grown in plastic pots containing a mixture of soil and vermiculite (3:1). The pots were then placed in an artificial growth chamber for five weeks. The growth conditions were as follows: the temperature was set to 24/16°C, the photoperiod was 16/8 h, and the relative humidity was 65–70%. Specific treatments were provided to the seedlings as follows: for heat and cold treatments, seedlings were exposed to 38°C and 4°C, respectively. For ABA and drought stress treatment, the seedlings were cultured in a nutrient solution medium with 100 *μ*M and 6000 PEG (w/v). All treatments were carried out in continuous time intervals of 1, 6, and 12 h, respectively, with biological triplicates. After that leaf samples were quickly frozen in liquid nitrogen and stored at −80°C for further use.

### 2.9. RNA Isolation and Transcriptional Profiling of BraPP2C under Various Stresses

Total RNA was isolated from the treated frozen leaves with Trizol (Invitrogen) following the manufacturer's instructions. RNA was reverse-transcribed into cDNA using the Primer Script RT reagent kit (TAKARA, Dalian China) according to their instructions. Specific primers were designed using Becan Designer 7.9 and are presented in Table S1. In order to check the specificity of the primers, we used the BLAST tool against the* B. rapa *genome for confirmation. RT-PCR was performed according to the guidelines of previous studies [41]. Relative fold expression was calculated with the comparative Ct-method. The expression patterns of all* BraPP2C* genes were analyzed based on a previous study [42]. Furthermore, gene expression levels were quantified by FPKM (fragments per kilobase of transcript per million fragments mapped) values, and heat maps were generated using an online omicshares tool (http://www.omicshare.com/).

## 3. Results

### 3.1. Characterization of* BraPP2C* Gene Family Members

In the present study, we identified 131 putative* BraPP2C* genes in* B. rapa* genome and were denoted as BraPP2C1 to BraPP2C131, based on phylogenetic analysis and their orthologous positions with* Arabidopsis*. The physicochemical feature of the* BraPP2C* proteins along with some key information of all the identified BraPP2C is provided in Table S2. The results of sequence analysis exhibited that the length of the BraPP2C proteins varied with a ranged from 102 (BraPP2C1) to 1465 (BraPP2C63) bp, with an average of 412.37 (aa). The detailed information of BraPP2C, including molecular weights (MW), theoretical isoelectric point (pI), the grand average of hydropathicity (GRAVY), and exon number ranged from 11.76 (BraPP2C27) to 160.43 (BraPP2C63) kDa, -0.777 (BraPP2C59) to 0.001 (BraPP2C41), 4.36 (BraPP2C85 to 9.37 (BraPP2C117), and 1 (BraPP2C73) to 21 (BraPP2C21), respectively. The significant variations reflect their functional diversity among BraPP2C, while the negative value of GRAVY further demonstrated that these proteins were unstable and hydrophilic in nature. Moreover, we analyzed the values of pI, GRAVY, and exon number, as shown in (Supplementary Figures 1, 2, and 3), respectively. The subcellular localization was predicated, and results proposed that most of the BraPP2C proteins were localized in mitochondria, cytoplasm, nuclei, chloroplasts, plasma membranes, endoplasmic reticulum, and vacuoles (Table S2). The gene structure analysis of BraPP2C revealed that the exon number of each* BraPP2C* gene ranged dramatically from 1 to 19 (Figure S4). Majority of* BraPP2C* genes consisted of only one exon; the rest of others showed significant variations, whereas BraPP2C32 possesses 19 exons, suggesting that both exon loss and gain occurred in* BraPP2C* gene family. Alongside, we systematically discovered the distribution of conserved motifs and their logos by online MEME server. A total of ten different consensus motifs were obtained in all of the BraPP2C proteins, and their distribution patterns are presented in Figures S5 and S6. Hence, during the evolutionary process, the BraPP2C demonstrated the extreme conservation within subgroups.

### 3.2. Chromosome Distribution and Syntenic Gene Collinearity Analysis

**Figure 1:**
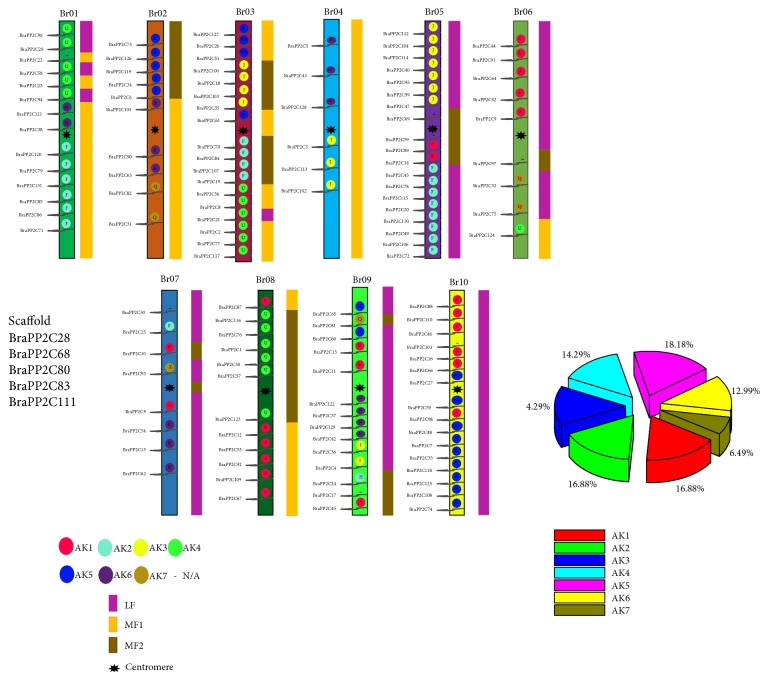
Chromosome locations of PP2C were obtained from the GFF file and displayed using Mapchart. The ancestral karyotypes are marked in different colors.

The physical chromosomal locations on* B. rapa*, across all ten chromosomes (Br01–Br10), were determined by the Mapchart software. The 126* BraPP2C* genes were dispersed across all ten chromosomes, ranging from 7 to 19 per chromosome, out of which 5 genes were found on the scaffold region (Figure 1). On each chromosome, the number of BraPP2C varies drastically; the largest number of BraPP2C family members was observed on chromosomes Br05 with 19 genes, followed by Br03 with 18 genes, whereas the least numbers were revealed on chromosomes Br04 and Br07; each contains 7 genes, respectively. Among these BraPP2C, five genes (*BraPP2C28*,* BraPP2C68*,* BraPP2C80*,* BraPP2C83*, and* BraPP2C111*) were present on the genomic scaffold. These results suggest the uneven distribution patterns of BraPP2C across ten different chromosomes. Based on a previously reported study, 24 ancestral genomic blocks (GBs) were reconstructed [27]. The color-coding for these block were slightly modified according to their position in a proposed ancestral karyotype (AK1-07) [27, 43]. The majority (18.18%) of* BraPP2C* genes were clustered in the AK5 region, followed by AK1 and AK2 (16.88%), whereas minimum genes (6.49%) were clustered in AK7 region (Figure 1).

**Figure 2:**
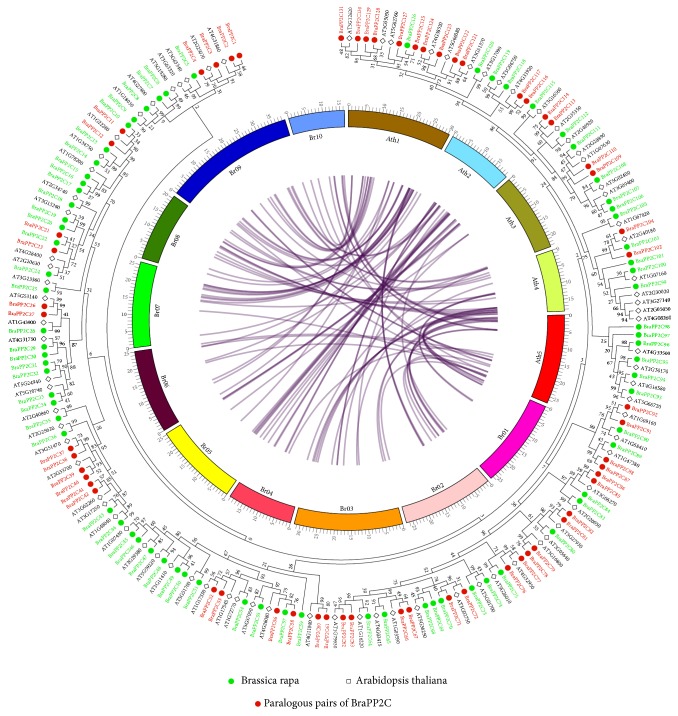
Phylogenetic relationship of PP2C between* B. rapa* and* A. thaliana*. The phylogenetic tree was constructed by MEGA 7 using the Maximum Likelihood Method (1000 bootstrap). Genes of PP2C paralog pairs are marked with red color. The collinear correlation for all genes of PP2C is displayed between* B. rapa* and* A. thaliana*. The 10 Chinese cabbage chromosomes (Br01–Br10) and five* A. thaliana* chromosomes (AT01–AT05) are shown in different random colors. The illustration was drawn using Circos Software.

The collinear relationships of the* PP2C* gene pairs between* Arabidopsis *and* B. rapa* are shown in Figure 2. Based on three subgenomes of* B. rapa*, LF contains more (42.86%)* BraPP2C* genes than the MF1 (32.77%) and MF2 (24.37%) subgenomes (Figure S7). In addition, the divergence time of the 29 paralogous pairs was estimated with the help of MEGA7.0 software by calculating the synonymous (*Ks*) and nonsynonymous substitution rates (*Ka*). The results proposed that most of the* BraPP2C* gene pairs represented less than 1.00 (*Ka/Ks)* ratios, suggesting the purifying selection of these genes except three pairs (*BraPP2C58*-*BraPP2C56*,* BraPP2C85*-*BraPP2C86*, and* BraPP2C125*-*BraPP2C127*) showed higher than 1.00 values, indicating positive selection. To estimate the divergence among these* BraPP2C* paralogous gene pairs, we calculated the values of* Ks* (Figure S8 and Table S3). The average divergence time among these paralogous is 12.37 MYA, suggesting that BraPP2C divergence occurred along with the* Arabidopsis* (9.6–16.1 MYA) [6].

### 3.3. Phylogenetic Analysis of* BraPP2C* Gene and Copy Number Variations/Gene Retention

To provide an overview of the evolutionary relationships among subgroup member of PP2C between* B. rapa* and* A. thaliana*, a comparative phylogenetic tree was constructed using MEGA7.0 software by adapting the maximum likelihood approach with 1000 bootstrap replications (Figure S9). The phylogenetic tree and domain composition further subcategorized the PP2C into thirteen subgroups: subgroup A-L and one unclassified. The results of the tree were consistent with the previously reported studies in* Arabidopsis* and rice [44]. Moreover, most of the BraPP2C clustered together with those from* Arabidopsis*, while 128 out of 131* BraPP2C* genes are further distributed among twelve subfamilies (A-L), and the remaining three (*BraPP2C35*,* BraPP2C60*, and* BraPP261*)* BraPP2C* genes cannot be grouped into any subfamilies and were categorized as unclassified. Most of the PP2C members were found in two subgroups E and F each with 25 genes, respectively, as compared to other subgroups. The complete overview and the distribution patterns among* Brassica rapa* and various ten species are described in Table 1.

Following the similar method, we also constructed a phylogenetic tree to compare the relationships of PP2C among* Arabidopsis, Amborella trichopoda, Vitis vinifera, Populus trichocarpa, Capsella rubella, Citrus sinensis, Carica papaya, Solanum lycopersicum, Fragaria vesca, Arabidopsis Iyarta, and* a bryophyte (*Physcomitrella patens*). Intriguingly, we observed diversification within subgroups of almost every species, which might indicate that different species used for tree topologies lead to slight variations (Supplementary Figures S10 and S11).

**Figure 3:**
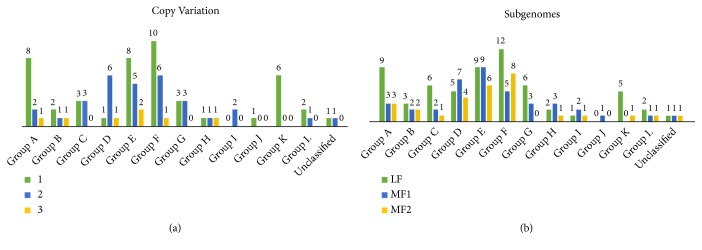
(a) and (b) The copy number variation and the ratio of PP2C among three subgenomes of* B. rapa*.

The copy number of variation and gene retention in* B. rapa* and* A. thaliana* during a* Brassica*-specific WGT event was also investigated. Meanwhile,* B. rapa *genome is based on three subgenomes and shares the same diploid ancestor with* A. thaliana *[26]. For each gene, we identified its syntenic paralogous and orthologous pairs by utilizing the* Brassica rapa* database (BRAD) (Table S4). In particular, we counted the retention of* BraPP2C* genes based on subgroups and by calculating the number of gene copies among three subgenomes. It was observed that the majority of genes were found either in a single, double or triple copy. Interestingly, for subgroup E, only two pairs were found with three copies and 6 each pair were identified for subgroup D and F with two copies, whereas the remaining did not show any copy variation and group F also shows the most number (25) of genes based on subgenomes comparison (Figures 3(a) and 3(b)). Our findings also revealed that gene retention exhibited almost with varied results, 15/15 subgroup A, 7/8 subgroup B, 9/9 subgroup C, 16/17 subgroup D, 24/25 subgroup E, 24/25 subgroup F, 9/9 subgroup G, 6/6 subgroup H, 4/4 subgroup I, 1/1 subgroup J, 6/7 subgroup K, 4/4 subgroup L, and 3/3 unclassified, respectively (Table S4).

### 3.4. Expression Pattern of BraPP2C in Various Tissues and the Correlation Networking Analysis of Paralogous Pairs

**Figure 4:**
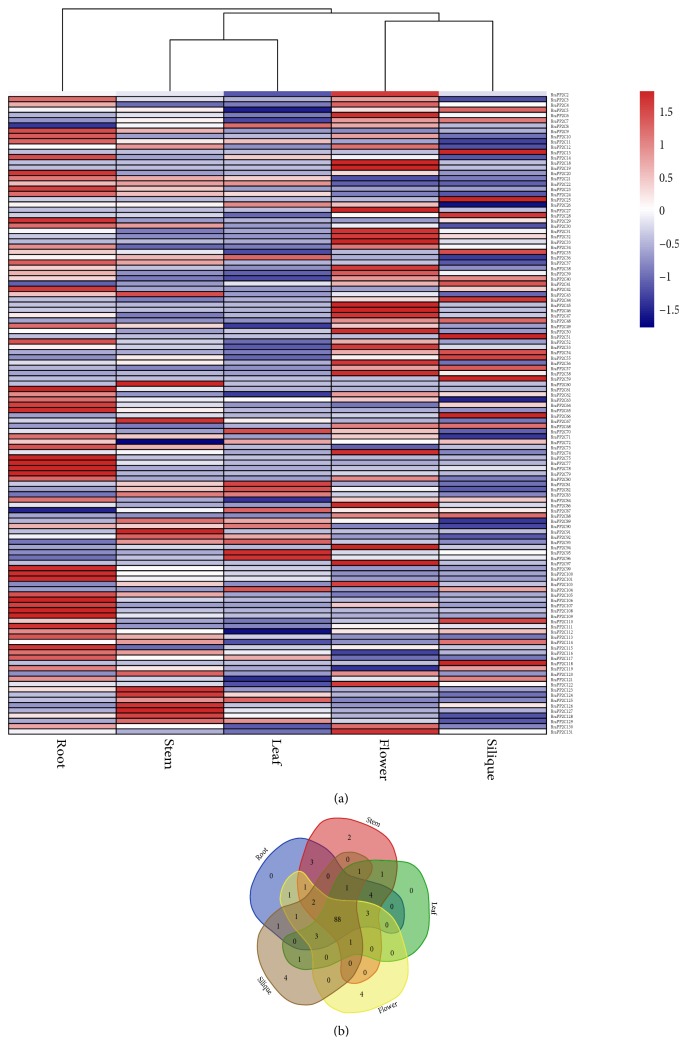
(a) and (b) Heatmap of expression profiles (in log_2_-based FPKM) for PP2C in the five various tissues: root, stem, leaf, flower, and silique. The expression levels are indicated by the color bar. (b). Venn diagram analysis of the tissue expression of PP2C.

To address the divergence and possible involvement of* BraPP2C* genes in* B. rapa* reflected in their growth and development. The trends of transcriptional profiles across five various tissues (roots, stems, leaves, flowers, and siliques) were determined based on previously published RNA-sequence data [45]. In this study, FPKM values were selected to represent* BraPP2C* genes expression and heat maps were constructed to demonstrate the relative transcriptional profiling of 131* BraPP2C* genes in various tissues. The results displayed high alterations in expression profiling among various subgroups members of PP2C in* B. rapa*. Among 131* BraPP2C* genes,* BraPP2C1*,* BraPP2C15*,* BraPP2C16*,* BraPP2C17*,* BraPP2C69*,* BraPP2C76*,* BraPP2C85*, and* BraPP2C98* showed no expression and* BraPP2C*,* BraPP2C18-19*,* BraPP2C27*,* BraPP2C51*,* BraPP2C59-60*,* BraPP2C91*,* BraPP2C97*, and* BraPP2C118* showed slight expression in one of any tissues (Figure 4(a); Table S5). The rest of the PP2C were expressed in at least two or more organs. However, few members of BraPP2C were specifically observed to be selectively expressed in tissue-specific clustering (Figure 4(b)), such that four of each gene in flowers and siliques were found and two of them were observed in the stem. Intriguingly, these genes indicated a possible role in organ development of* B. rapa.*

We next investigated the expression trends among 29 paralogous pairs for* PP2C* genes along with calculated their Pearson's correlation coefficient values (Figure S12A; Table S6). These paralogous pairs exhibited significant variation in various tissues. The results showed that more than 20 pairs were highly expressed in all tissues and 13 pairs with higher PCC values (>0.6). Meanwhile, four pairs (BraPP2C2_BraPP2C1, BraPP2C77_BraPP2C76, BraPP2C85_BraPP2C86, and BraPP2C104_BraPP2C102) showed no correlation values, and four pairs (BraPP2C42_BraPP2C41, BraPP2C60_BraPP2C61, BraPP2C66_BraPP2C67, and BraPP2C72_BraPP2C71) exhibited a negative correlation. Also, the clustering image did not show any variability among various tissues (Figure S12B). Intriguingly, these results indicated that a possible involvement of* BraPP2C* genes during growth by showing an overlapping in expression pattern might be the basis of crosstalk for signal transductions pathways and plant improvements. Despite the divergence in the expression profiles for BraPP2C between paralogous pairs, suggesting that after duplication in the evolutionary process, some of these pairs may acquire new functions.

### 3.5. Analysis of Putative Regulatory* Cis*-Element in BraPP2C and Coregulatory Expression Network Analysis

**Figure 5:**
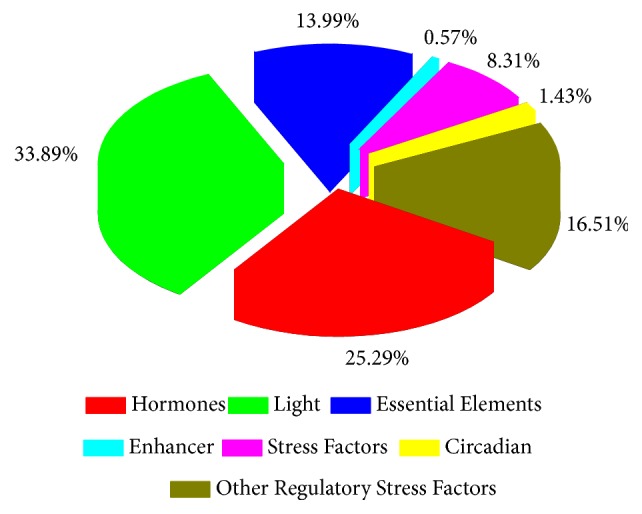
The ratios of different cis-elements.

The* cis*-elements in promoter regions are closely associated with gene transcription, and their response to stress as gene expression is mediated through interaction between* cis*-regulatory elements and its cognate transcription factors [46]. Therefore, 2.0 kb upstream sequences were downloaded from* B. rapa* database and analyzed using the PlantCARE database [37]. In the promoter regions of BraPP2C, we identified a total of 7 common* cis*-regulatory elements (Figure 5 and Table S7). The skn-1 motif was responsive for endosperm expression. MBS is deliberated as an MYB binding site, which is involved in drought-inducibility. DRE and LTR are* cis*-acting elements that are known to control functions during adverse conditions, such as dehydration, chilling, and salt stress. Few of the* cis*-regulatory elements were actively receptive against hormonal stresses, such as ethylene, gibberellin, auxin, and salicylic acid. Circadian* cis*-regulatory element regulates the circadian rhythm of the plant system. As shown in Figure 5, the participation of hormones, light, essential elements, enhancers, stress factors, circadian, and other regulatory stress factors was higher in* BraPP2C* genes. As a consequence, the data suggests the presence of a large number of conserved* cis*-regulatory elements that are crucial in mediating responses to adverse environmental stresses or stress-related hormones. Though, to prove such assumptions, a further validation step is required. Moreover, these results highlighted the stress-responsive disposition of* BraPP2C* genes.

**Figure 6:**
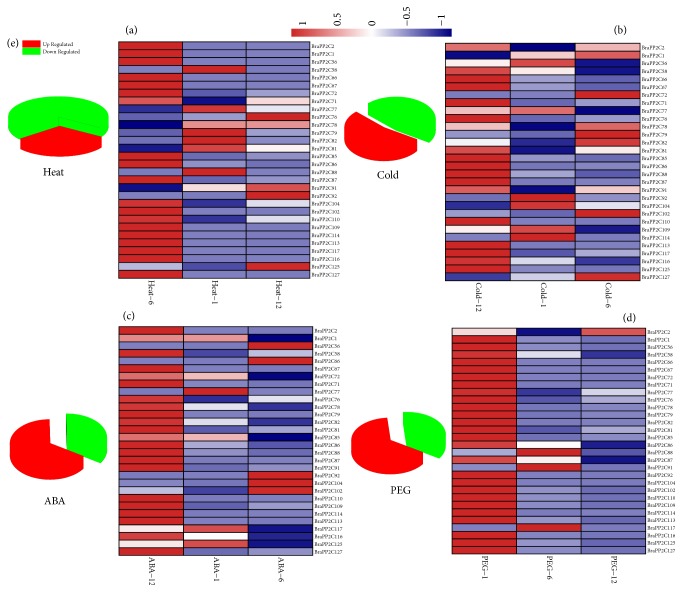
Expression analysis of* PP2C* genes under heat, cold, ABA, and drought stress treatments in* B. rapa*.

**Figure 7:**
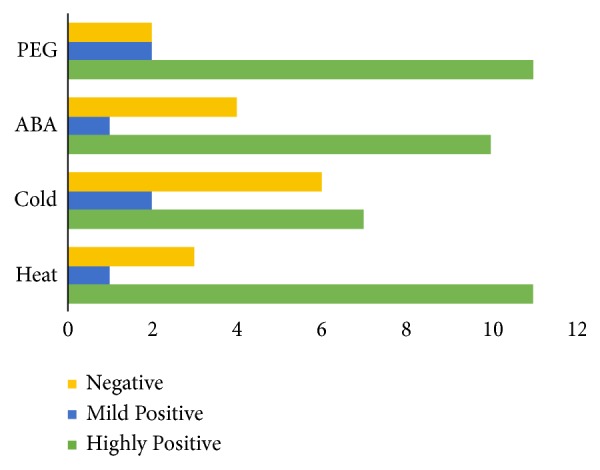
Pearson's correlation coefficient (PCC) values under response to heat, cold, ABA, and drought treatments.

For functional studies, gene expression profiling provides valuable clues, many* PP2C *have been implicated in plants against abiotic stress conditions, and the presence of stress-responsive* cis*-elements in the promoter region further suggests their possible involvement in* B. rapa* response to different environmental stimuli. Therefore, we investigated the transcriptional profiles of 15 PP2C paralogous gene pairs of* B. rapa* subjected to cold, heat, PEG, and ABA treatments using qRT-PCR analysis (Figure 6; Table S8). Our results indicated that the* BraPP2C* genes showed diversity in their expression level against four various treatments. Interestingly, the expression patterns of some genes induced rapidly when exposed to ABA and drought application. In contrary, some genes were highly upregulated or downregulated by either of one or two stresses. Under heat treatment, a dominant portion of the* BraPP2C* genes showed highly fluctuated transcriptional profiling levels, including 30% upregulated and 70% downregulated genes. The exposure of* B. rapa* to cold stress resulted in approximately 54% of upregulated genes. The transcriptional level was significantly increased under ABA and drought treatments; most of the genes were highly 57% and 64% expressed, respectively. Taken together, the diverse expression pattern of* BraPP2C* genes may suggest various roles after exposure to abiotic and hormone stress conditions. To understand the correlation and coregulatory network among these paralogous pairs, we calculated Pearson's correlation coefficient (PCC) values based on their relative expression data. For the correlation network, we make three categories concerning PCC values, such as more than 0.6 (Highly Positive), less than 0.5 but greater than 0 (Mild Positive), and negative values with (Negative) correlation (Figure 7 and Table S9). Heat and drought stress showed highly positive and closer relationship by showing (11) PCC values each, while cold stress was found with a high number of negative (6) PCC values, suggesting its different nature among the paralogous pairs of* BraPP2C *genes.

Moreover, we examined the interaction network of BraPP2C proteins with the help of STRING software. The BraPP2C proteins were highly correlated with each other in the interaction network. As shown in Figure S13, most of the subgroups of BraPP2C exhibited a dense network when compared with each other. Most of the proteins were highly located in the center and only a few of them did not interact with others, which indicated the complexity of the interaction network. Therefore, this PP2C may involve in the regulation of many downstream/upstream gene factors by playing a crucial role in fundamental molecular mechanisms of plants.

## 4. Discussion

**Figure 8:**
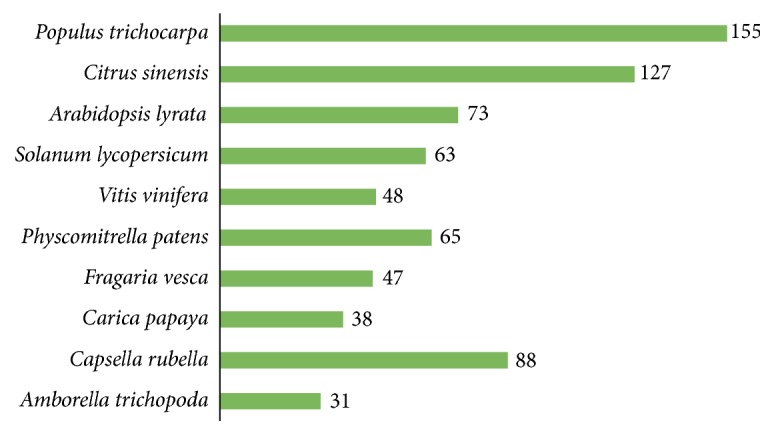
The proportion of* PP2C* genes among various species.

In the plant kingdom, the* PP2C* gene family is considered as one of the largest families and has been characterized in several crop species by utilizing bioinformatics strategies. Previously, various members of* PP2C* genes were identified in maize [47], rice [48],* Arabidopsis *[49], hot pepper [50], banana [51], and* Brachypodium distachyon* [3]. In this study, a comprehensive genome-wide analysis was performed, including gene identification, phylogenetic relationships, evolutionary analysis, chromosomal localizations, conserved domain motifs, and gene structure organization analysis. In addition, gene expression patterns of some key* BraPP2C* genes were also determined under heat, cold, ABA, and drought stresses. Herein, a total of 131* BraPP2C* genes were identified. The* PP2C* genes were further categorized into 11, 12, or 13 subfamilies depending on the plant species according to evolutionary analysis. The proportion of PP2C members was much higher as compared to lower plants. For this reason, we further isolated the* PP2C* genes in ten various species and calculated their number as well, such as* Amborella trichopoda *which contains 31* PP2C* genes,* Vitis vinifera *(48)*, Populus trichocarpa *(155)*, Capsella rubella *(88)*, Citrus sinensis *(127)*, Carica papaya *(38)*, Solanum lycopersicum *(63)*, Fragaria vesca *(47)*, Arabidopsis lyrata *(73), and* Physcomitrella patens* (65) as shown in Figure 8 and Table S10. As a consequence, a high degree of divergence was observed between* PP2C* genes from lower plants to higher plants, suggesting that it may be correlated with the complex adaptation to environmental stimuli [52]. Overall, the number of* PP2C* genes has remained distinct in each investigated species, proposing that variation in evolutionary patterns is occurring, due to gradual changes in rounds of whole-genome duplication (WGD) and subsequent gene losses/gains by natural selection constraints.

Notably, gene duplication, being the predominant influencing force for broad expansion of the gene family, upgrades its biological function and evolutionary processes [53]. Thus, to study gene family fractionation, a linkage between them and their morphotypes,* B. rapa* is an excellent model plant because it has experienced WGD and an additional WGT event [26, 27]. Considering the importance of evolutionary analysis, most of the angiosperms have undergone either one or multiple polyploidization events [54–56]. As a result, for duplicated genes, it provides opportunities to diverge in various evolutionary ways. Subsequently, each of these genes experienced one of the following three evolutionary fates: subfunctionalization (the ancestral function is subdivided between copies), neofunctionalization (one copy acquires a new function), or nonfunctionalization (one copy becomes unexpressed or functionless) [57]. Furthermore, we predicted the pressure of natural selection by calculating the synonymous (*Ks*) and nonsynonymous substitution rates (*Ka*) and were identified by the MEGA7.0 program. Also, during evolutionary processes and expansion of genes family, these indicators are used for the selection history. If the value of* Ka/Ks* is lower than 1.00, it represents purifying selection,* Ka/Ks* = 1 means neutral selection, and* Ka/Ks*> 1 means positive selection [7]. In our work, we estimated the divergence time among the 29 paralogous pairs of* BraPP2C* genes. Majority of the BraPP2C paralogous pairs showed less than 1.00* Ka/Ks* ratios, speculating the purifying selection of these paralogous pairs. On the other hand, only three pairs show more than 1.00 values, suggesting positive selection. Moreover, the divergence time of these paralogous pairs of the* BraPP2C* genes ranged from 0.03 to 0.84 (*Ks* values) with an average mean divergence of 12.37 (MYA). Notably, the divergence time of* BraPP2C* paralogous gene pairs was 12.37 MYA, which further intimates that their divergence occurred during the* Brassica *and* Arabidopsis *duplication (9.6–16.1 MYA) [7].

Plants productivity encounters a severe threat by maintaining the optimum crop production, due to a wide range of environmental challenges. Surrounded by various stress factors, such as heat, cold, ABA, and drought represent the major constraint for agricultural crop production. In regulating plant' tolerance to multiple abiotic stress factor a well-known hormone, ABA plays a crucial role. However, in* B*.* rapa*, the role of the core components of ABA signaling against* PP2C* genes in responding to various stress conditions is mainly obscure. Specifically, various members of PP2C group A (ABI1, ABI2, HAB1, HAB2, AHG1, and PP2CA) resulted into increase the ABA sensitivity after double and triple mutation, suggesting the diverse outcome in ABA signaling [17, 58–61]. Previous studies reported that* PP2C* regulates positively against salt tolerance in* Arabidopsis* and drought in peach [62, 63] to modulate the stress severity. In* Arabidopsis*, two members of* PP2C* genes were responded differently, such as* AP2C1* expression was strongly induced by cold, drought, and wounding, but* AP2C2* was slightly influenced by these treatments [64]. These findings highlighted the significance of critical members of the* PP2C* gene family in the model plant* Arabidopsis*, but their specific functions may be significantly varied in* B. rapa.* In the present study, various* PP2C* genes showed high striking transcriptional changes followed by heat, cold, ABA, and drought stresses, indicating that some of these genes might be pivotal to stress tolerance in* B. rapa*. Some of the recent advances in the functional dissection of PP2C candidate genes uncovered their importance in the life cycle of* Arabidopsis* and rice [48, 65], although their associated roles in* B. rapa *are mostly unnoticed. Aiming for achieving gene expression patterns in various growth phases of* BraPP2C*, we utilized the previously reported RNA-sequence data and analyzed the expression profile of* PP2C* genes in various tissues (root, stem, leaf, flower, and silique). Results demonstrated that most of the* BraPP2C* genes showed a varying response to multiple tissues and some of the genes were highly expressed in all the tissues or in some cases; it was negative. However, few genes have shown tissue-specific expression, such that four genes were commonly identified in roots and flower and two of them were expressed in the stem as well, intimating their importance in plant development. In addition, gene family expansion and the duplication types in the neofunctionalization or subfunctionalization models are mainly associated with tissue expression divergence [66–68]. In* BraPP2C* genes, we also explored the promoter regions for identification of common conserved* cis*-regulatory elements. As to further elucidate the predicated and the possible functions of* BraPP2C *genes in transcriptional regulation, the result represented significant variation among* BraPP2C* genes and was mostly responsive to both biotic-abiotic factors and plant hormones stresses.

It is indispensable to perceive the fundamental molecular mechanisms of plants for the adaptability of stress tolerance and enhancement in crop yield under adverse stress conditions. Therefore, our outcome of the study provides valuable insight and will serve as a basis for exploring the pivotal role of* BraPP2C* genes in the plant against abiotic stresses. It may also assist to reveal the potential functions of* BraPP2C* genes in response to both abiotic and hormone stress conditions.

## 5. Conclusions

In this study, we identified a total of 131* BraPP2C *gene family members by genome-wide analysis in* Brassica rapa *genome. A comprehensive analysis was carried out to study the phylogenetic relationships, evolutionary analysis, exon-intron organization, chromosomal localization, protein structural features, interaction network analysis, and conserved motifs. According to the phylogenetic relationships, the BraPP2C were classified into thirteen subgroups. Moreover, transcriptional profiling revealed BraPP2C candidate genes might have participated in the plant stress tolerance particularly to heat, cold, ABA, and drought stress. These* BraPP2C* genes can be utilized to facilitate by functionally characterize them, laying the foundation for elucidating their specific regulatory mechanisms and ultimately applying them in molecular breeding programs of this important vegetable crop. In summary, the integration of our findings has provided a novel insight and unique features of* BraPP2C *genes that may play a potential role in mediating regulation of signal transduction and particularly under various stress conditions.

## Figures and Tables

**Table 1:** The distribution of *PP2C* genes family is various species.

Subgroup of PP2C	BraPP2C	AthPP2C	AtrPP2C	CrPP2C	CpPP2C	FvPP2C	PpPP2C	VvPP2C	SlPP2C	AlPP2C	CsPP2C	PtPP2C
A	15	10	2	10	4	6	0	5	6	11	11	11
B	7	6	1	4	3	1	0	0	3	2	6	5
C	10	7	4	8	3	4	0	5	5	6	18	13
D	16	9	4	11	6	8	4	9	12	9	15	29
E	25	12	4	16	6	8	14	5	9	13	18	26
F	25	13	6	16	3	4	0	8	8	12	23	25
G	9	6	3	8	2	7	0	5	6	6	12	14
H	6	3	3	4	3	3	28	3	5	3	3	15
I	4	2	0	3	2	1	19	2	3	2	10	4
J	1	2	0	1	0	0	0	0	1	1	0	1
K	6	3	1	4	3	3	0	3	2	4	5	7
L	4	0	2	1	3	1	0	2	2	2	6	4
Unclassified	3	3	1	2	0	1	0	1	1	2	0	2

*∗BraPP2C= Brassica rapa, AthPP2C= Arabidopsis thaliana, AtrPP2C= Amborella trichopoda, CrPP2C= Capsella rubella, CpPP2C= Carica papaya, FvPP2C= Fragaria vesca, PpPP2C= Physcomitrella patens, VvPP2C= Vitis vinifera, SlPP2C= Solanum lycopersicum, AlPP2C= Arabidopsis lyrata, CsPP2C= Citrus sinensis, and PtPP2C= Populus trichocarpa.*

## Data Availability

The data used to support the findings of this study are available in this manuscript and supporting materials.

## Supplementary Materials

Supplementary Materials Figure S1: indication of isoelectric point (pI) values for PP2C based on* B. rapa* chromosomes (Br01-Br10). Figure S2: grand average of hydropathicity (GRAVY) of* PP2C *genes. Figure S3: the number of exon for the* PP2C* gene based on* B. rapa* chromosomes (Br01-Br10). Figure S4: gene structure, the exon-intron, and upstream/downstream region are represented by black boxes and blue boxes, respectively. At the bottom of the figure, the relative position is proportionally displayed based on the kilobase scale. Figure S5: the conserved motifs of PP2C were elucidated by MEME. Different motifs and their positions are represented by different colors, numbered 1–10 at the bottom. Figure S6: the LOGOS of PP2C were also elucidated by MEME online server. Figure S7: the ratios of three subgenomes of* B. rapa *for* PP2C* genes. Figure S8: the correlation between* Ks* and* Ka* for paralogous gene pairs. Figure S9: phylogenetic relationship of PP2C between* B. rapa* and* A. thaliana*. The phylogenetic tree was constructed by MEGA 7 using the Maximum Likelihood Method (1000 bootstrap). Genes of various subgroups of PP2C are marked with different color. Figure S10: phylogenetic relationship of PP2C among other species and* A. thaliana*. The phylogenetic tree was constructed by MEGA 7 using the Maximum Likelihood Method (1000 bootstrap). Genes of PP2C for different subgroups are marked with different colors. Figure S11: phylogenetic relationship of PP2C among other species and* A. thaliana*. The phylogenetic tree was constructed by MEGA 7 using the Maximum Likelihood Method (1000 bootstrap). Genes of PP2C for different subgroups are marked with different colors. Figure S12A: heatmap of expression profiles for 29 paralogous pairs in five various tissues: root, stem, leaf, flower, and silique. The Pearson correlation coefficients (PCCs) are also displayed in brackets, while NA indicates no available results for PCC. B. Venn diagram analysis of the tissue expression of paralogous pairs for PP2C. Figure S13: the coregulatory network for PP2C presented by the STRING server. Table 1. Sequences of the* BraPP2C* gene primers used for quantitative real-time PCR. Table 2: the basic information of* PP2C* genes identified in* Brassica rapa. *Table 3: the gene with outlier* Ka/Ks* values and estimated divergence time for syntenic pairs of BraPP2C. Table 4: identification of BraPP2C syntenic genes between* A. thaliana* along with three subgenomes of* Brassica rapa*. Table 5: the FPKM values of* PP2C* genes in* B. rapa*. Table 6: syntenic paralog pairs of BraPP2C with PC and FPKM values. Table 7: cis-elements of* PP2C* genes in* Brassica rapa*. Table 8: relative expression pattern of* BraPP2C* genes along with PC values with respect to various treatments by qRT-PCR. Table 9: the Pearson's correlation coefficient (PCC) of the stress-induced* PP2C* genes in* Brassica rapa. *Table 10: information of* PP2C* genes in 10 plants in this work.
