# Supplementary material for: Genome-Wide Identification, Evolution, and Transcriptional Profiling of PP2C Gene Family in Brassica rapa
Source: Biomed Res Int. 2019 Apr 3;2019:2965035. doi: 10.1155/2019/2965035 (PMC6470454; doi:10.1155/2019/2965035)
Supplement: Supplementary Materials — Figure S1: indication of isoelectric point (pI) values for PP2C based on B. rapa chromosomes (Br01-Br10). Figure S2: grand average of hydropathicity (GRAVY) of PP2C genes. Figure S3: the number of exon for the PP2C gene based on B. rapa chromosomes (Br01-Br10). Figure S4: gene structure, the exon-intron, and upstream/downstream region are represented by black boxes and blue boxes, respectively. At the bottom of the figure, the relative position is proportionally displayed based on the kilobase scale. Figure S5: the conserved motifs of PP2C were elucidated by MEME. Different motifs and their positions are represented by different colors, numbered 1–10 at the bottom. Figure S6: the LOGOS of PP2C were also elucidated by MEME online server. Figure S7: the ratios of three subgenomes of B. rapa for PP2C genes. Figure S8: the correlation between Ks and Ka for paralogous gene pairs. Figure S9: phylogenetic relationship of PP2C between B. rapa and A. thaliana. The phylogenetic tree was constructed by MEGA 7 using the Maximum Likelihood Method (1000 bootstrap). Genes of various subgroups of PP2C are marked with different color. Figure S10: phylogenetic relationship of PP2C among other species and A. thaliana. The phylogenetic tree was constructed by MEGA 7 using the Maximum Likelihood Method (1000 bootstrap). Genes of PP2C for different subgroups are marked with different colors. Figure S11: phylogenetic relationship of PP2C among other species and A. thaliana. The phylogenetic tree was constructed by MEGA 7 using the Maximum Likelihood Method (1000 bootstrap). Genes of PP2C for different subgroups are marked with different colors. Figure S12A: heatmap of expression profiles for 29 paralogous pairs in five various tissues: root, stem, leaf, flower, and silique. The Pearson correlation coefficients (PCCs) are also displayed in brackets, while NA indicates no available results for PCC. B. Venn diagram analysis of the tissue expression of paralogous pairs for PP2C. Figure S13: t [file 2965035.f1.zip › Merged Supplementary Figures (1).pdf]

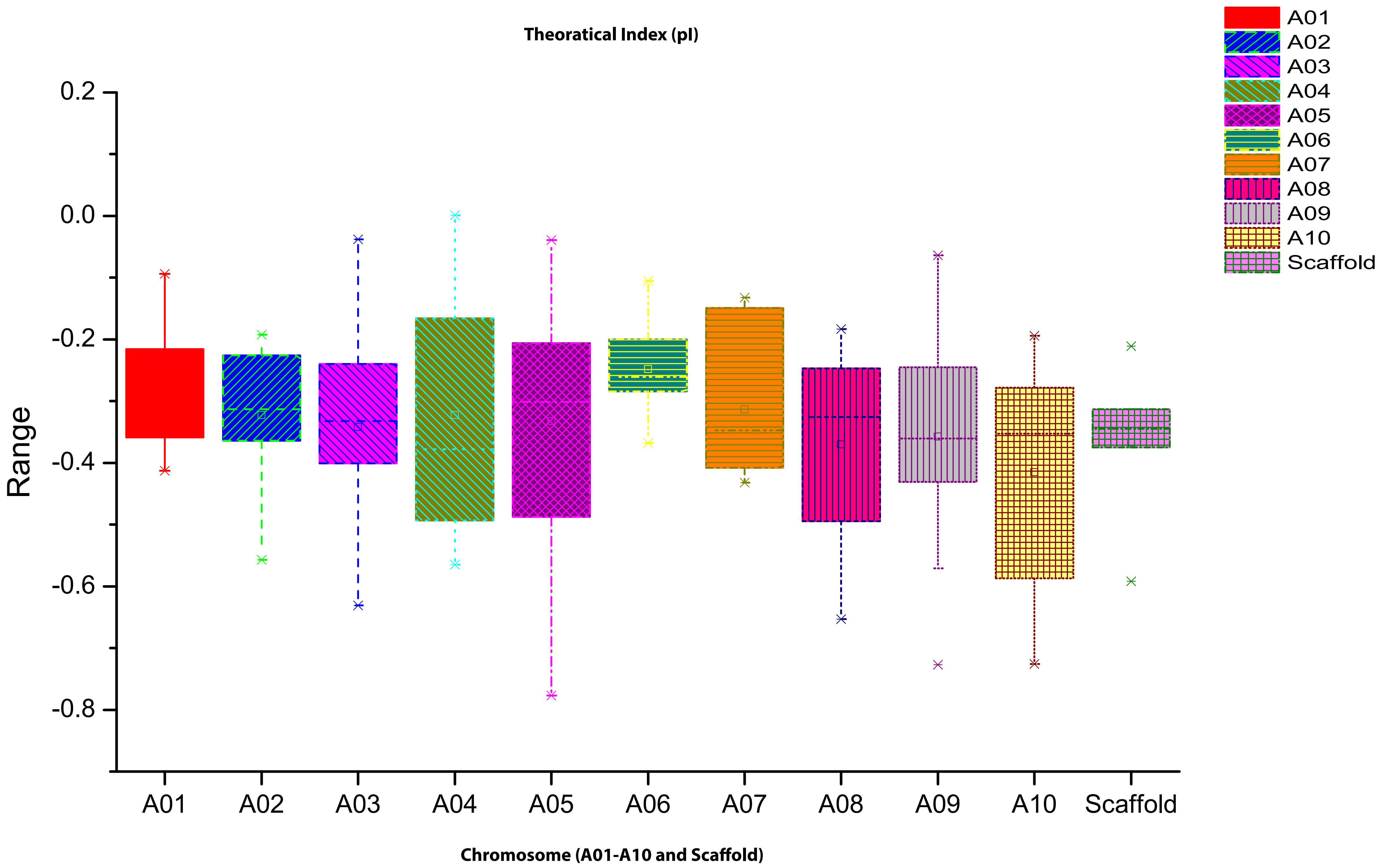

Theoratical Index (pI)
Range
0.2
0.0
-0.2
-0.4
-0.6
-0.8
A01
A02
A03
A04
A05
A06
A07
A08
A09
A10
Scaffold
Chromosome (A01-A10 and Scaffold)

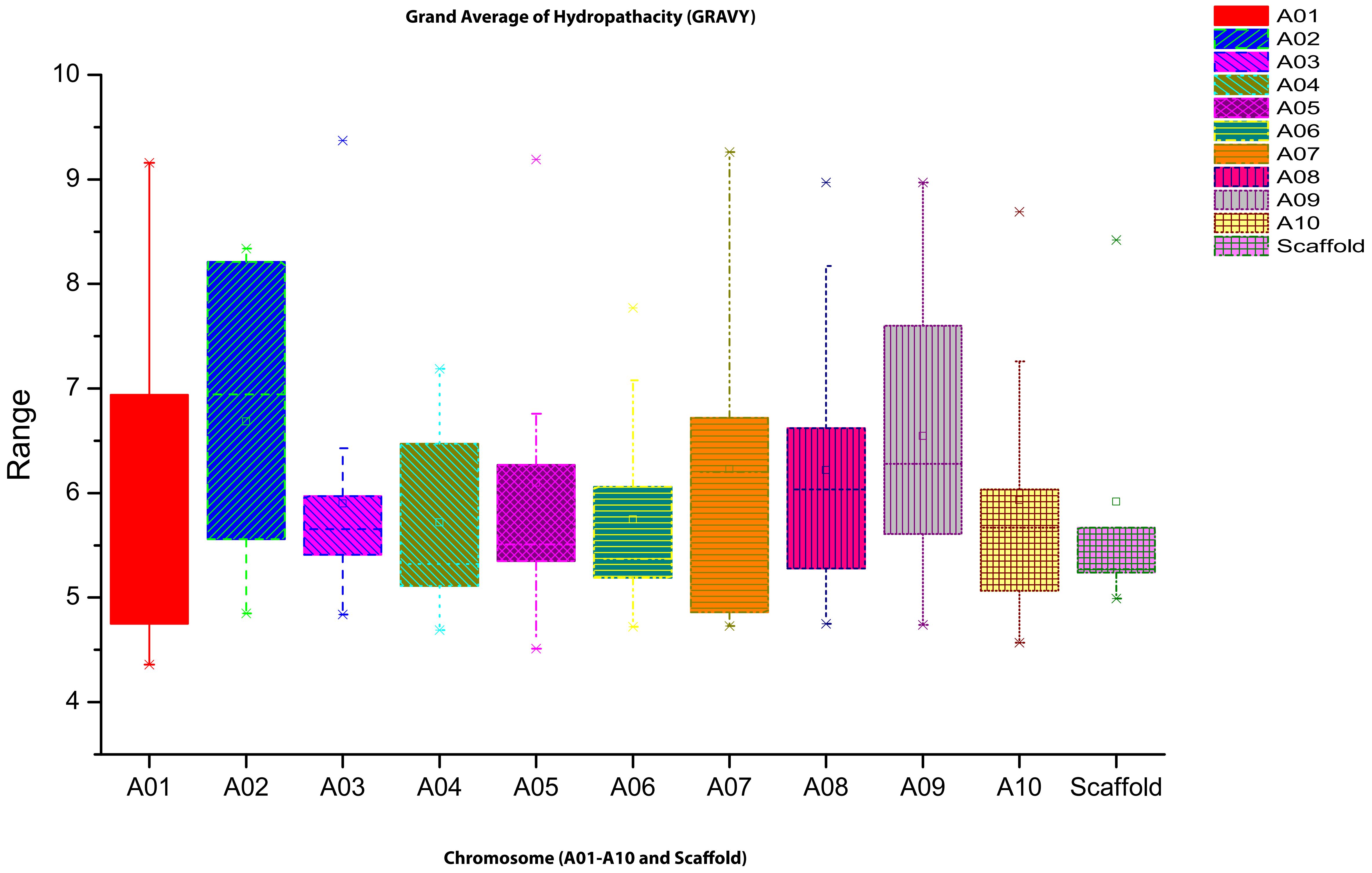

Grand Average of Hydropathacity (GRAVY)
Range
10
9
8
7
6
5
4
A01
A02
A03
A04
A05
A06
A07
A08
A09
A10
Scaffold
Chromosome (A01-A10 and Scaffold)

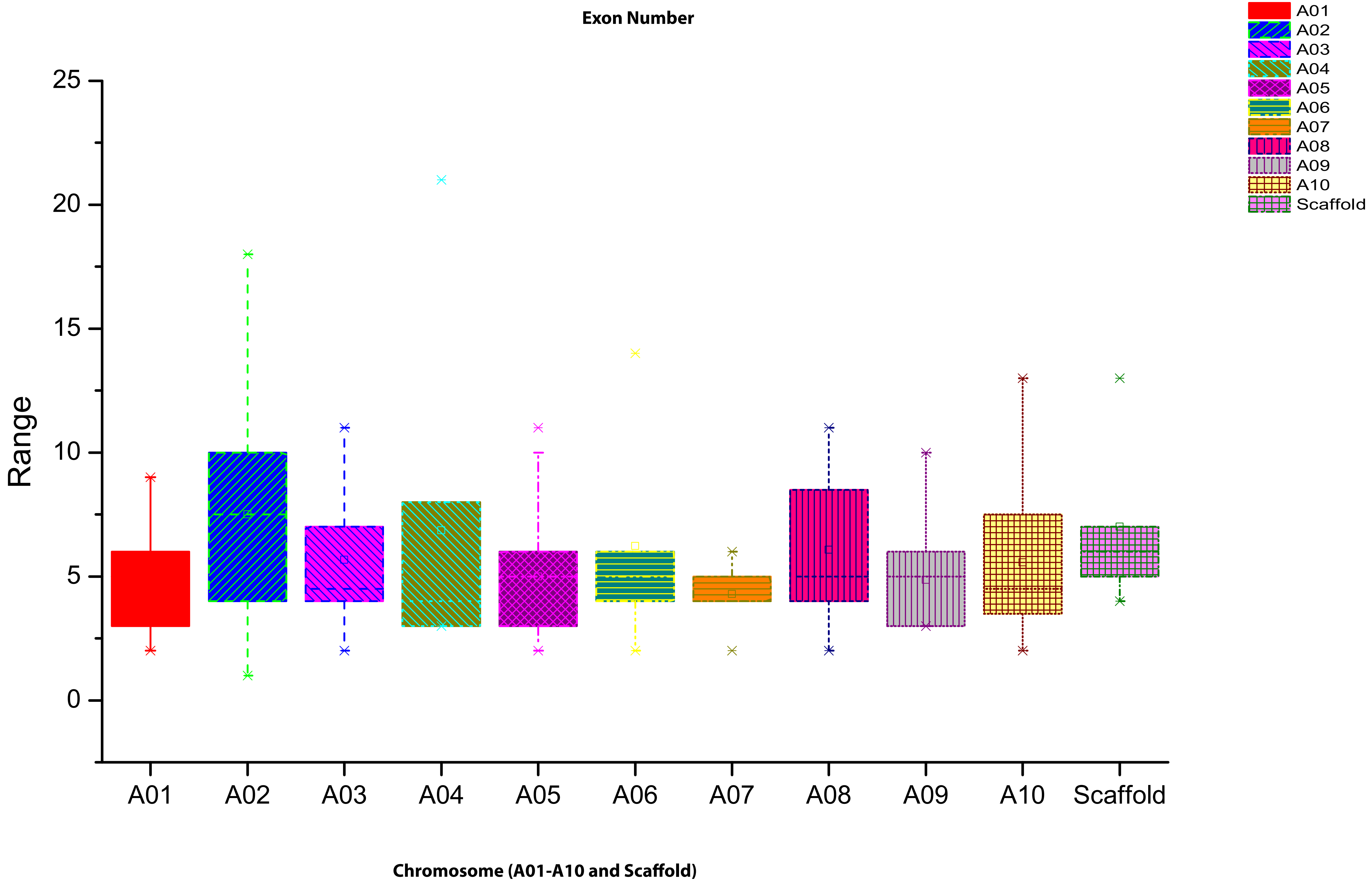

Exon Number
Range
25
20
15
10
5
0
A01
A02
A03
A04
A05
A06
A07
A08
A09
A10
Scaffold
Chromosome (A01-A10 and Scaffold)

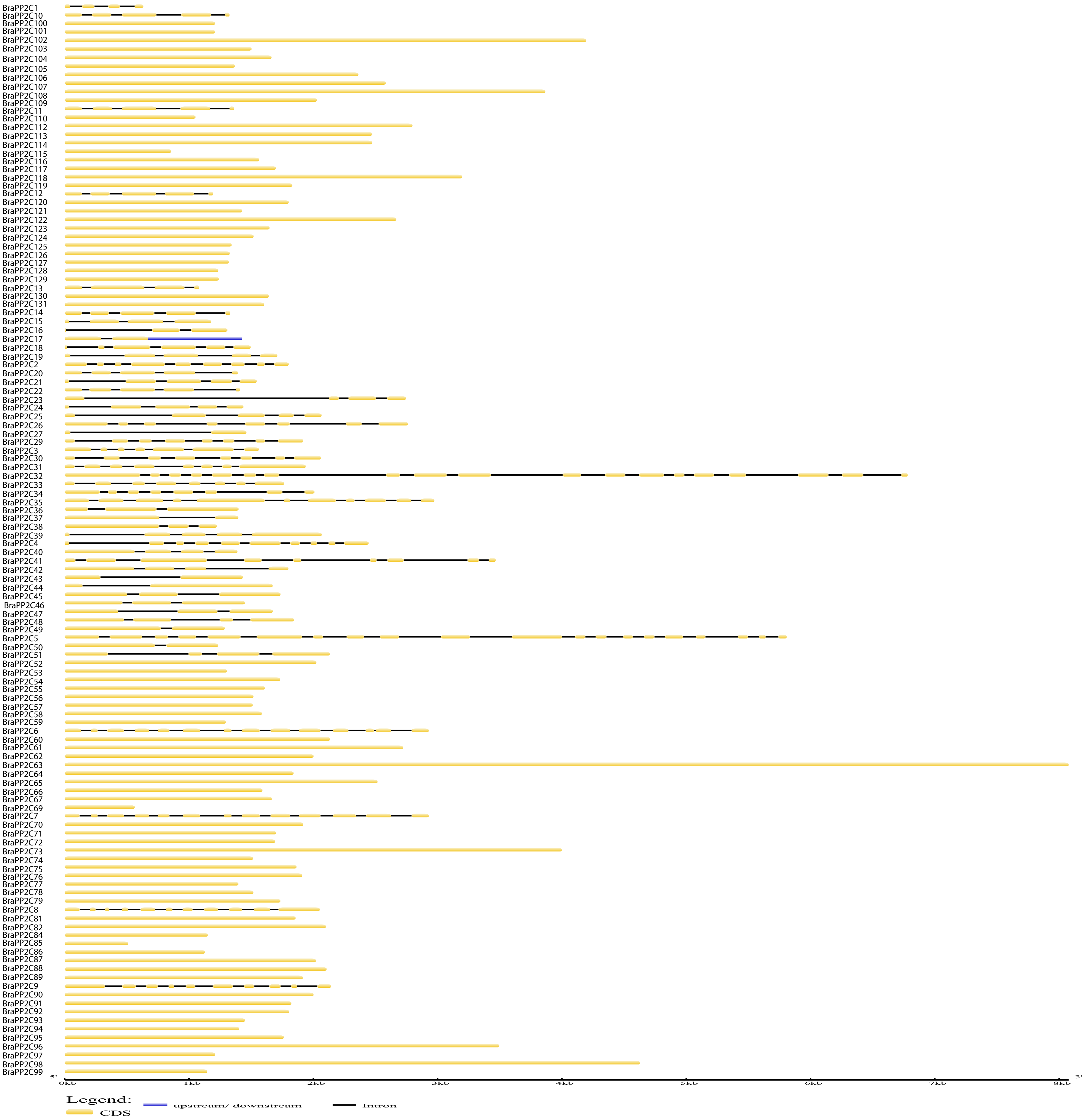
BraPP2C1
BraPP2C10
BraPP2C100
BraPP2C101
BraPP2C102
BraPP2C103
BraPP2C104
BraPP2C105
BraPP2C106
BraPP2C107
BraPP2C108
BraPP2C109
BraPP2C11
BraPP2C110
BraPP2C112
BraPP2C113
BraPP2C114
BraPP2C115
BraPP2C116
BraPP2C117
BraPP2C118
BraPP2C119
BraPP2C12
BraPP2C120
BraPP2C121
BraPP2C122
BraPP2C123
BraPP2C124
BraPP2C125
BraPP2C126
BraPP2C127
BraPP2C128
BraPP2C129
BraPP2C13
BraPP2C130
BraPP2C131
BraPP2C14
BraPP2C15
BraPP2C16
BraPP2C17
BraPP2C18
BraPP2C19
BraPP2C2
BraPP2C20
BraPP2C21
BraPP2C22
BraPP2C23
BraPP2C24
BraPP2C25
BraPP2C26
BraPP2C27
BraPP2C29
BraPP2C3
BraPP2C30
BraPP2C31
BraPP2C32
BraPP2C33
BraPP2C34
BraPP2C35
BraPP2C36
BraPP2C37
BraPP2C38
BraPP2C39
BraPP2C4
BraPP2C40
BraPP2C41
BraPP2C42
BraPP2C43
BraPP2C44
BraPP2C45
BraPP2C46
BraPP2C47
BraPP2C48
BraPP2C49
BraPP2C5
BraPP2C50
BraPP2C51
BraPP2C52
BraPP2C53
BraPP2C54
BraPP2C55
BraPP2C56
BraPP2C57
BraPP2C58
BraPP2C59
BraPP2C6
BraPP2C60
BraPP2C61
BraPP2C62
BraPP2C63
BraPP2C64
BraPP2C65
BraPP2C66
BraPP2C67
BraPP2C69
BraPP2C7
BraPP2C70
BraPP2C71
BraPP2C72
BraPP2C73
BraPP2C74
BraPP2C75
BraPP2C76
BraPP2C77
BraPP2C78
BraPP2C79
BraPP2C8
BraPP2C81
BraPP2C82
BraPP2C84
BraPP2C85
BraPP2C86
BraPP2C87
BraPP2C88
BraPP2C89
BraPP2C9
BraPP2C90
BraPP2C91
BraPP2C92
BraPP2C93
BraPP2C94
BraPP2C95
BraPP2C96
BraPP2C97
BraPP2C98
BraPP2C99
5'
0kb
1kb
2kb
3kb
4kb
5kb
6kb
7kb
8kb
3'
Legend:
upstream/ downstream
Intron
CDS

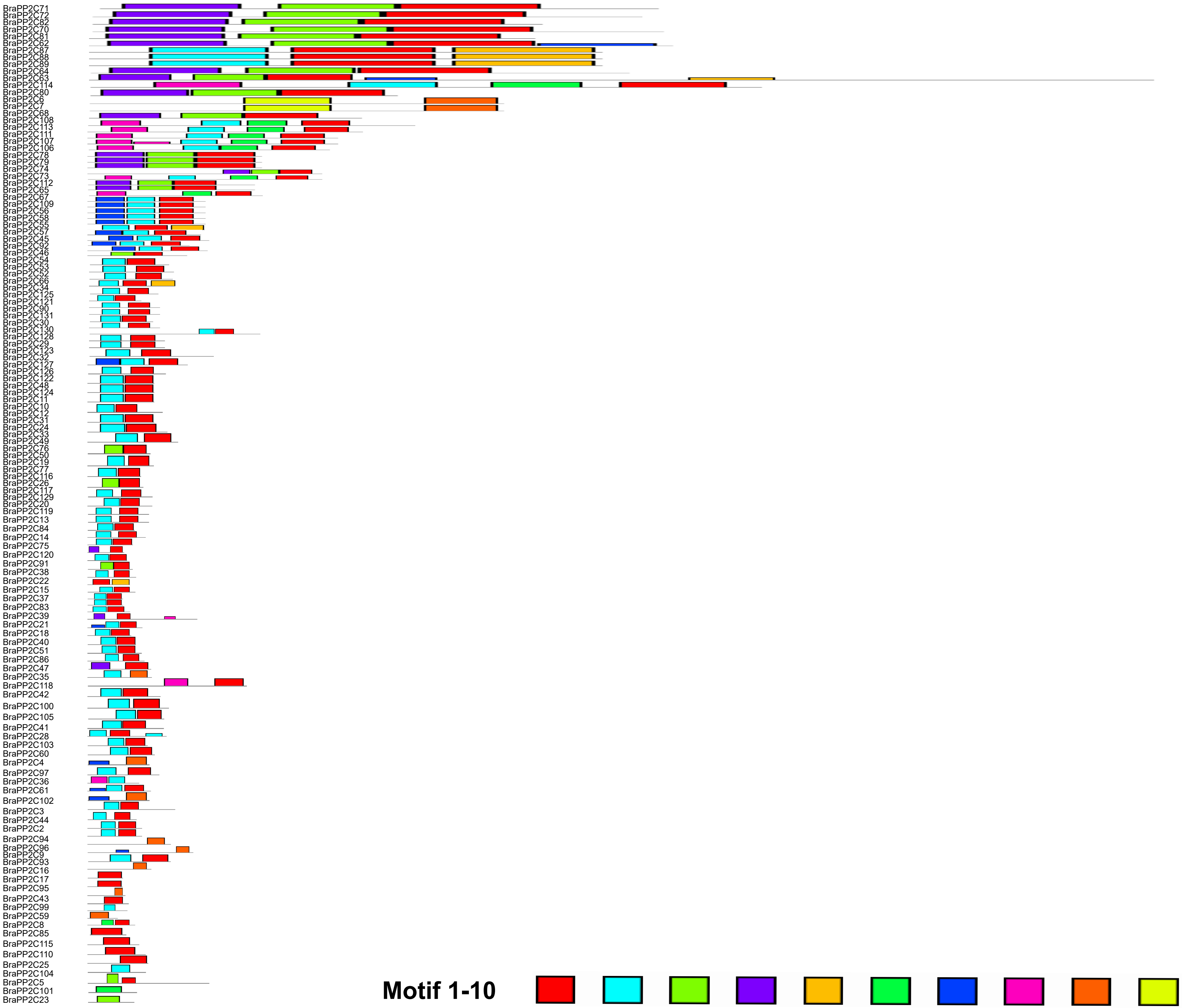
BraPP2C71
BraPP2C72
BraPP2C82
BraPP2C70
BraPP2C81
BraPP2C62
BraPP2C87
BraPP2C88
BraPP2C89
BraPP2C64
BraPP2C63
BraPP2C114
BraPP2C80
BraPP2C6
BraPP2C7
BraPP2C68
BraPP2C108
BraPP2C113
BraPP2C111
BraPP2C107
BraPP2C106
BraPP2C78
BraPP2C79
BraPP2C74
BraPP2C73
BraPP2C112
BraPP2C65
BraPP2C67
BraPP2C109
BraPP2C56
BraPP2C58
BraPP2C55
BraPP2C57
BraPP2C45
BraPP2C92
BraPP2C46
BraPP2C54
BraPP2C53
BraPP2C52
BraPP2C66
BraPP2C34
BraPP2C125
BraPP2C121
BraPP2C90
BraPP2C131
BraPP2C30
BraPP2C130
BraPP2C128
BraPP2C29
BraPP2C123
BraPP2C32
BraPP2C127
BraPP2C126
BraPP2C122
BraPP2C48
BraPP2C124
BraPP2C11
BraPP2C10
BraPP2C12
BraPP2C31
BraPP2C24
BraPP2C33
BraPP2C49
BraPP2C76
BraPP2C50
BraPP2C19
BraPP2C77
BraPP2C116
BraPP2C26
BraPP2C117
BraPP2C129
BraPP2C20
BraPP2C119
BraPP2C13
BraPP2C84
BraPP2C14
BraPP2C75
BraPP2C120
BraPP2C91
BraPP2C38
BraPP2C22
BraPP2C15
BraPP2C37
BraPP2C83
BraPP2C39
BraPP2C21
BraPP2C18
BraPP2C40
BraPP2C51
BraPP2C86
BraPP2C47
BraPP2C35
BraPP2C118
BraPP2C42
BraPP2C100
BraPP2C105
BraPP2C41
BraPP2C28
BraPP2C103
BraPP2C60
BraPP2C4
BraPP2C97
BraPP2C36
BraPP2C61
BraPP2C102
BraPP2C3
BraPP2C44
BraPP2C2
BraPP2C94
BraPP2C96
BraPP2C9
BraPP2C93
BraPP2C16
BraPP2C17
BraPP2C95
BraPP2C43
BraPP2C99
BraPP2C59
BraPP2C8
BraPP2C85
BraPP2C115
BraPP2C110
BraPP2C25
BraPP2C104
BraPP2C5
BraPP2C101
BraPP2C23
Motif 1-10

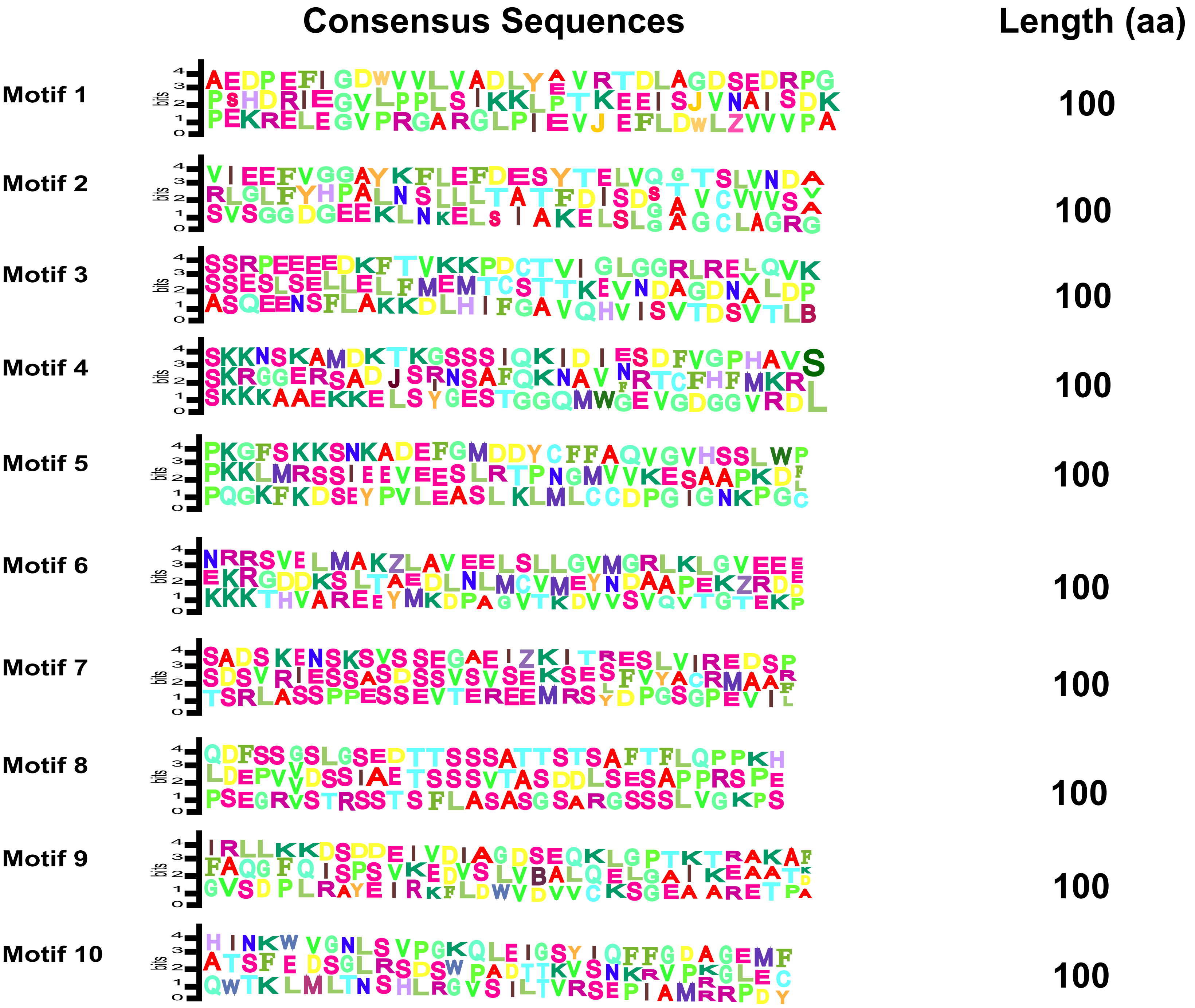

Consensus Sequences
Length (aa)
Motif 1
100
Motif 2
100
Motif 3
100
Motif 4
100
Motif 5
100
Motif 6
100
Motif 7
100
Motif 8
100
Motif 9
100
Motif 10
100

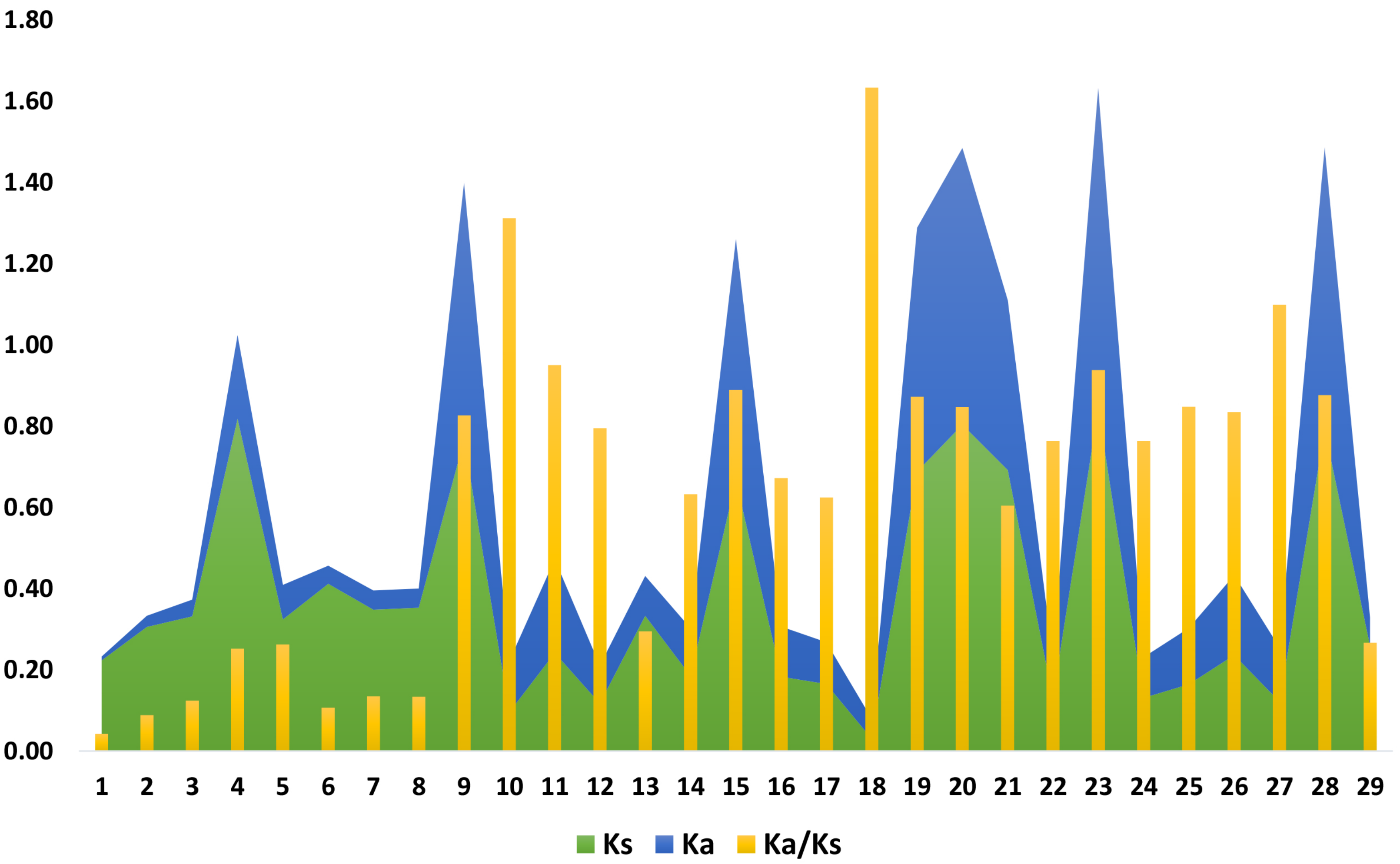
1.80
1.60
1.40
1.20
1.00
0.80
0.60
0.40
0.20
0.00
1 2 3 4 5 6 7 8 9 10 11 12 13 14 15 16 17 18 19 20 21 22 23 24 25 26 27 28 29
Ks
Ka
Ka/Ks

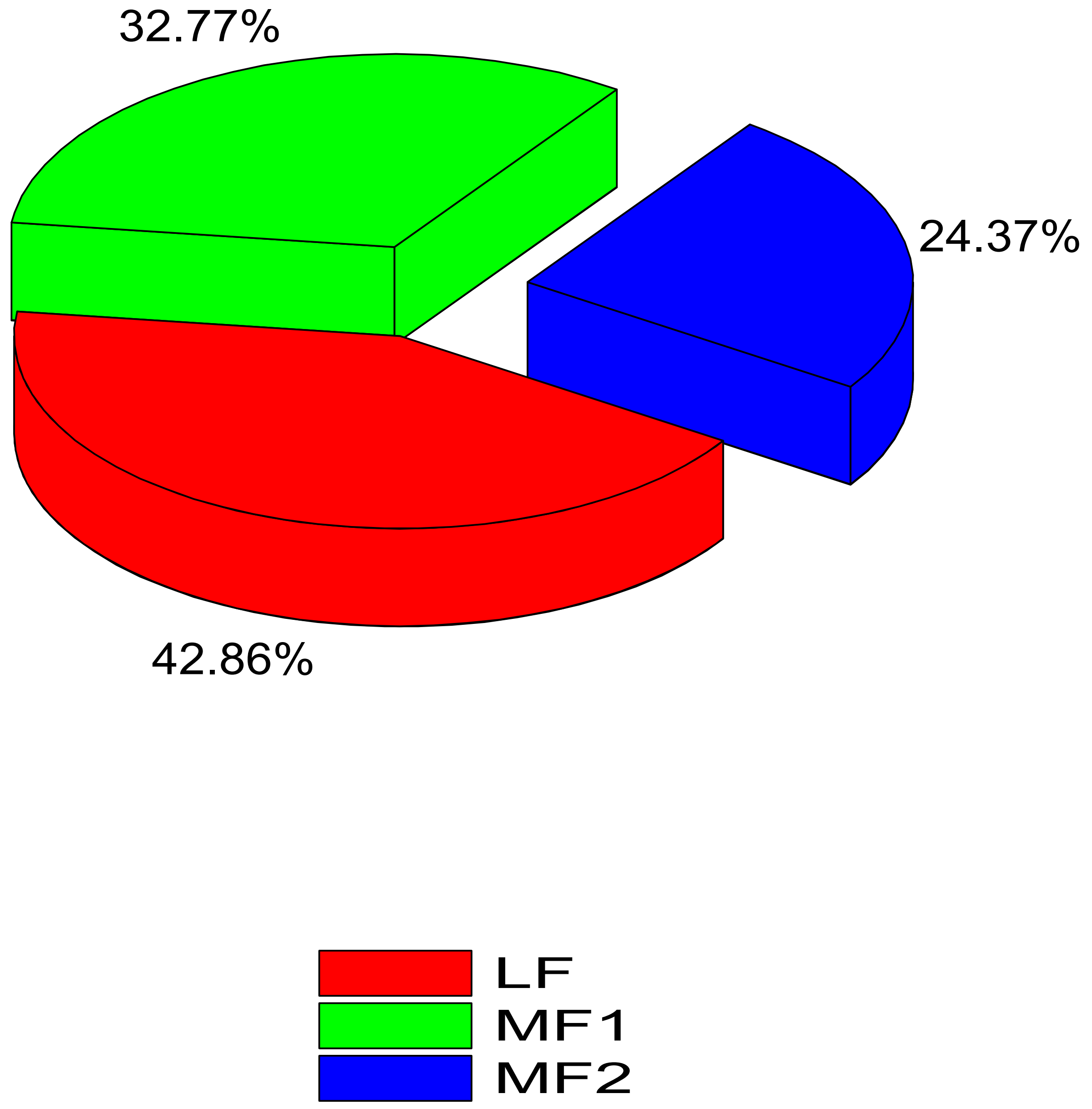
32.77%
24.37%
42.86%
LF
MF1
MF2

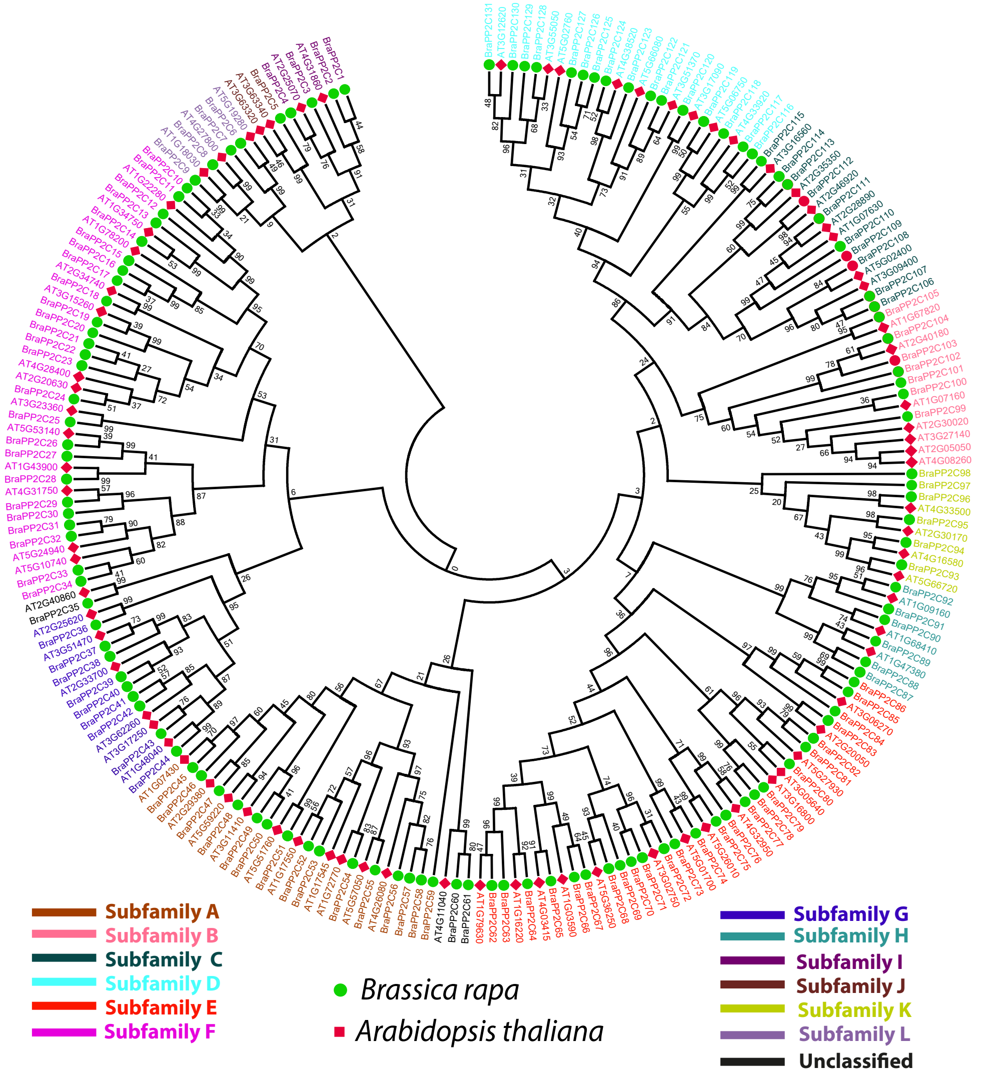
BraPP2C1
BraPP2C2
AT4G31860
BraPP2C3
AT2G25070
BraPP2C4
BraPP2C5
AT3G63340
AT3G63320
AT5G19280
BraPP2C6
BraPP2C7
AT4G27800
BraPP2C8
AT1G18030
BraPP2C9
BraPP2C10
BraPP2C11
AT1G22280
BraPP2C12
AT1G34750
BraPP2C13
BraPP2C14
AT1G78200
BraPP2C15
BraPP2C16
BraPP2C17
AT2G34740
BraPP2C18
AT3G15260
BraPP2C19
BraPP2C20
BraPP2C21
BraPP2C22
BraPP2C23
AT4G28400
AT2G20630
BraPP2C24
AT3G23360
BraPP2C25
AT5G53140
BraPP2C26
BraPP2C27
AT1G43900
BraPP2C28
AT4G31750
BraPP2C29
BraPP2C30
BraPP2C31
BraPP2C32
AT5G24940
AT5G10740
BraPP2C33
BraPP2C34
AT2G40860
BraPP2C35
AT2G25620
BraPP2C36
AT3G51470
BraPP2C37
BraPP2C38
AT2G33700
BraPP2C39
BraPP2C40
BraPP2C41
BraPP2C42
AT3G62260
AT3G17250
BraPP2C43
AT1G48040
BraPP2C44
AT1G07430
BraPP2C45
BraPP2C46
AT2G29380
BraPP2C47
AT5G59220
BraPP2C48
AT3G11410
BraPP2C49
BraPP2C50
AT5G51760
BraPP2C51
AT1G17550
BraPP2C52
BraPP2C53
AT1G17545
AT1G72770
BraPP2C54
AT5G57050
BraPP2C55
AT4G26080
BraPP2C56
BraPP2C57
BraPP2C58
BraPP2C59
AT4G11040
BraPP2C60
BraPP2C61
AT1G79630
BraPP2C62
BraPP2C63
AT1G16220
BraPP2C64
AT4G03415
BraPP2C65
AT1G03590
BraPP2C66
BraPP2C67
AT5G36250
BraPP2C68
BraPP2C69
BraPP2C70
BraPP2C71
AT3G02750
BraPP2C72
BraPP2C73
AT5G01700
AT5G26010
BraPP2C74
BraPP2C75
AT4G32950
BraPP2C76
BraPP2C77
BraPP2C78
BraPP2C79
AT3G16800
AT3G05640
BraPP2C80
AT5G27930
BraPP2C81
BraPP2C82
AT2G20050
BraPP2C83
BraPP2C84
AT3G06270
BraPP2C85
BraPP2C86
BraPP2C87
BraPP2C88
AT1G47380
BraPP2C89
AT1G68410
BraPP2C90
BraPP2C91
AT1G09160
BraPP2C92
AT5G66720
BraPP2C93
AT4G16580
BraPP2C94
AT2G30170
BraPP2C95
AT4G33500
BraPP2C96
BraPP2C97
BraPP2C98
AT4G08260
AT2G05050
AT3G27140
AT2G30020
BraPP2C99
AT1G07160
BraPP2C100
BraPP2C101
BraPP2C102
BraPP2C103
AT2G40180
BraPP2C104
AT1G67820
BraPP2C105
BraPP2C106
BraPP2C107
AT3G09400
AT5G02400
BraPP2C108
BraPP2C109
BraPP2C110
AT1G07630
BraPP2C111
AT2G28890
BraPP2C112
AT2G46920
BraPP2C113
AT2G35350
BraPP2C114
AT3G16560
BraPP2C115
BraPP2C116
BraPP2C117
AT4G33920
BraPP2C118
AT5G06750
BraPP2C119
AT3G17090
BraPP2C120
AT3G51370
BraPP2C121
BraPP2C122
AT5G66080
BraPP2C123
AT4G38520
BraPP2C124
BraPP2C125
BraPP2C126
BraPP2C127
AT5G02760
AT3G55050
BraPP2C128
BraPP2C129
BraPP2C130
AT3G12620
BraPP2C131
Subfamily A
Subfamily B
Subfamily C
Subfamily D
Subfamily E
Subfamily F
Brassica rapa
Arabidopsis thaliana
Subfamily G
Subfamily H
Subfamily I
Subfamily J
Subfamily K
Subfamily L
Unclassified

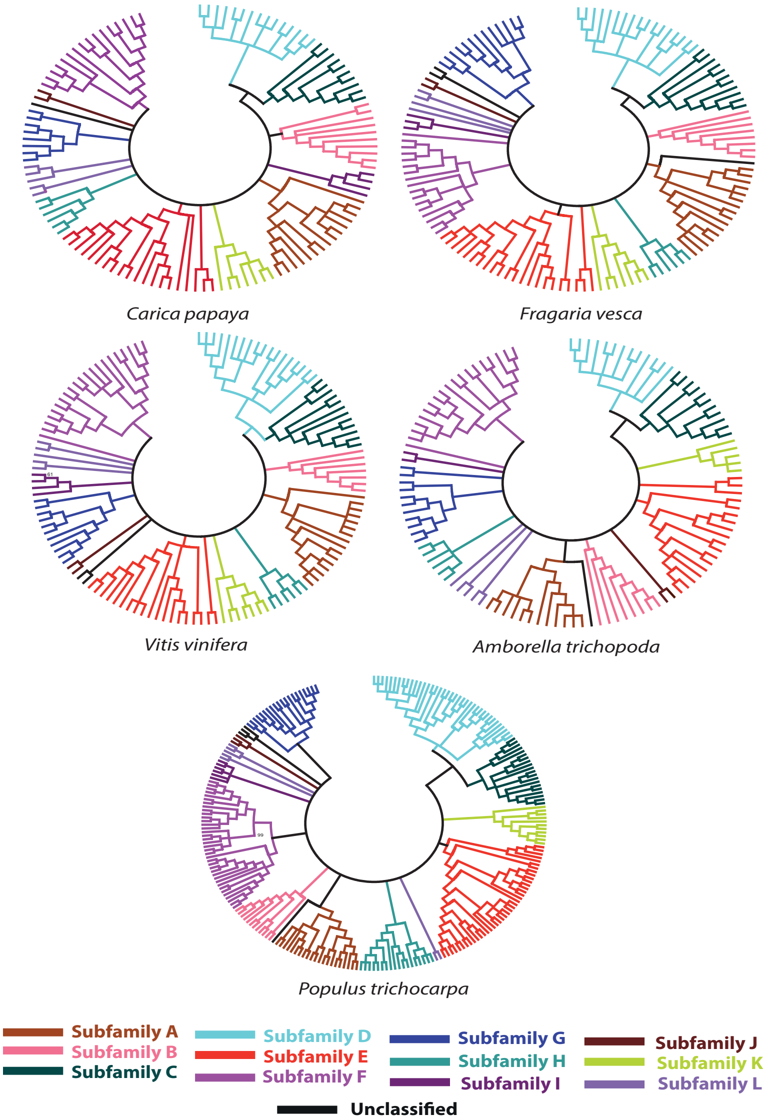

Carica papaya
Fragaria vesca
61
Vitis vinifera
Amborella trichopoda
99
Populus trichocarpa
Subfamily A
Subfamily B
Subfamily C
Subfamily D
Subfamily E
Subfamily F
Subfamily G
Subfamily H
Subfamily I
Subfamily J
Subfamily K
Subfamily L
Unclassified

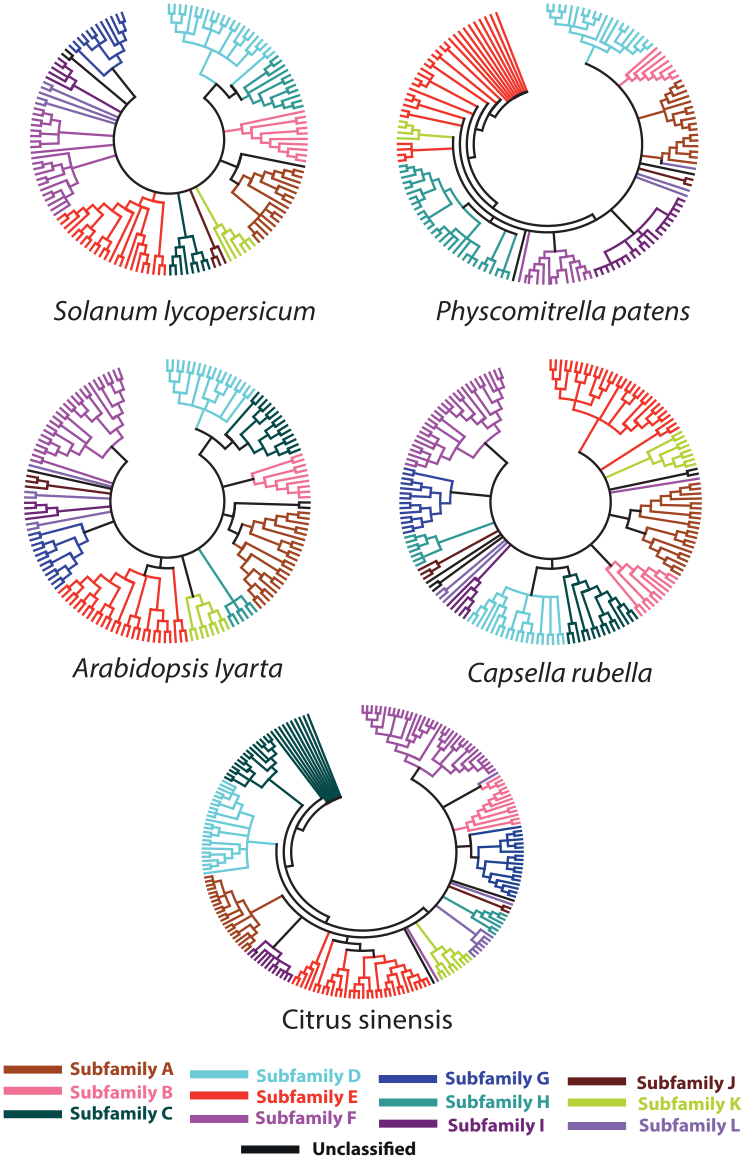
Solanum lycopersicum
Physcomitrella patens
Arabidopsis lyarta
Capsella rubella
Citrus sinensis
Subfamily A
Subfamily B
Subfamily C
Subfamily D
Subfamily E
Subfamily F
Subfamily G
Subfamily H
Subfamily I
Subfamily J
Subfamily K
Subfamily L
Unclassified

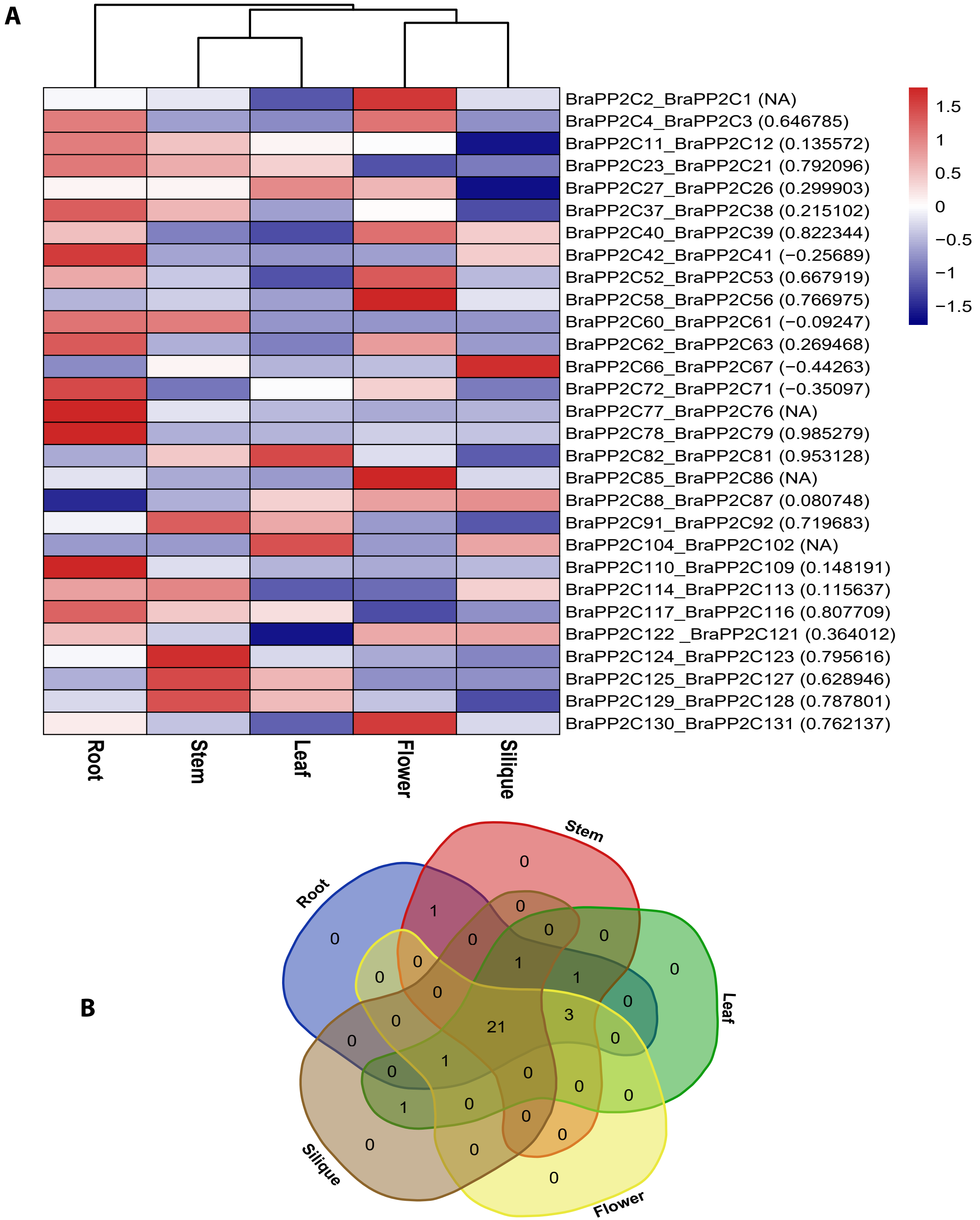
A
BraPP2C2_BraPP2C1 (NA)
BraPP2C4_BraPP2C3 (0.646785)
BraPP2C11_BraPP2C12 (0.135572)
BraPP2C23_BraPP2C21 (0.792096)
BraPP2C27_BraPP2C26 (0.299903)
BraPP2C37_BraPP2C38 (0.215102)
BraPP2C40_BraPP2C39 (0.822344)
BraPP2C42_BraPP2C41 (−0.25689)
BraPP2C52_BraPP2C53 (0.667919)
BraPP2C58_BraPP2C56 (0.766975)
BraPP2C60_BraPP2C61 (−0.09247)
BraPP2C62_BraPP2C63 (0.269468)
BraPP2C66_BraPP2C67 (−0.44263)
BraPP2C72_BraPP2C71 (−0.35097)
BraPP2C77_BraPP2C76 (NA)
BraPP2C78_BraPP2C79 (0.985279)
BraPP2C82_BraPP2C81 (0.953128)
BraPP2C85_BraPP2C86 (NA)
BraPP2C88_BraPP2C87 (0.080748)
BraPP2C91_BraPP2C92 (0.719683)
BraPP2C104_BraPP2C102 (NA)
BraPP2C110_BraPP2C109 (0.148191)
BraPP2C114_BraPP2C113 (0.115637)
BraPP2C117_BraPP2C116 (0.807709)
BraPP2C122 _BraPP2C121 (0.364012)
BraPP2C124_BraPP2C123 (0.795616)
BraPP2C125_BraPP2C127 (0.628946)
BraPP2C129_BraPP2C128 (0.787801)
BraPP2C130_BraPP2C131 (0.762137)
Root
Stem
Leaf
Flower
Silique
1.5
1
0.5
0
−0.5
−1
−1.5
B
Stem
Root
Leaf
Silique
Flower

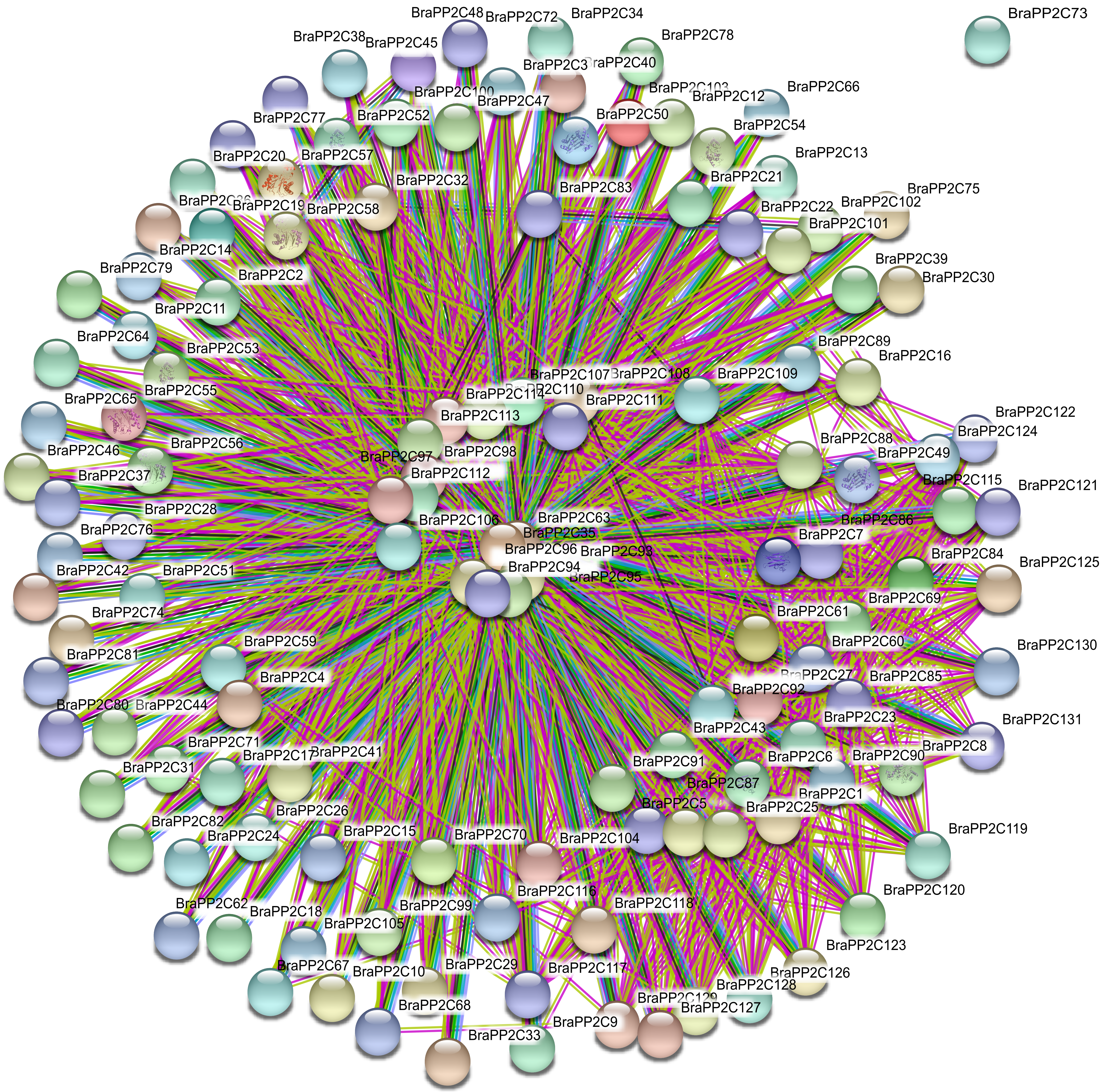
BraPP2C73
BraPP2C48
BraPP2C72
BraPP2C34
BraPP2C38
BraPP2C45
BraPP2C78
BraPP2C3
BraPP2C100
BraPP2C66
BraPP2C12
BraPP2C77
BraPP2C52
BraPP2C47
BraPP2C50
BraPP2C54
BraPP2C20
BraPP2C57
BraPP2C13
BraPP2C21
BraPP2C32
BraPP2C19
BraPP2C58
BraPP2C83
BraPP2C75
BraPP2C22
BraPP2C102
BraPP2C101
BraPP2C14
BraPP2C2
BraPP2C79
BraPP2C39
BraPP2C30
BraPP2C11
BraPP2C64
BraPP2C53
BraPP2C89
BraPP2C16
BraPP2C107
BraPP2C108
BraPP2C109
BraPP2C65
BraPP2C55
BraPP2C114
BraPP2C111
BraPP2C113
BraPP2C122
BraPP2C124
BraPP2C46
BraPP2C56
BraPP2C88
BraPP2C49
BraPP2C97
BraPP2C98
BraPP2C37
BraPP2C112
BraPP2C115
BraPP2C121
BraPP2C28
BraPP2C106
BraPP2C63
BraPP2C86
BraPP2C76
BraPP2C35
BraPP2C7
BraPP2C96
BraPP2C93
BraPP2C84
BraPP2C42
BraPP2C51
BraPP2C94
BraPP2C125
BraPP2C69
BraPP2C74
BraPP2C61
BraPP2C59
BraPP2C81
BraPP2C60
BraPP2C130
BraPP2C4
BraPP2C85
BraPP2C92
BraPP2C80
BraPP2C44
BraPP2C43
BraPP2C23
BraPP2C131
BraPP2C71
BraPP2C17
BraPP2C41
BraPP2C8
BraPP2C6
BraPP2C90
BraPP2C91
BraPP2C31
BraPP2C87
BraPP2C1
BraPP2C5
BraPP2C25
BraPP2C26
BraPP2C82
BraPP2C24
BraPP2C15
BraPP2C70
BraPP2C104
BraPP2C119
BraPP2C120
BraPP2C116
BraPP2C62
BraPP2C18
BraPP2C99
BraPP2C118
BraPP2C105
BraPP2C123
BraPP2C67
BraPP2C10
BraPP2C29
BraPP2C117
BraPP2C126
BraPP2C128
BraPP2C127
BraPP2C68
BraPP2C9
BraPP2C33
